# The effects of oxidative stress on female reproduction: a review

**DOI:** 10.1186/1477-7827-10-49

**Published:** 2012-06-29

**Authors:** Ashok Agarwal, Anamar Aponte-Mellado, Beena J Premkumar, Amani Shaman, Sajal Gupta

**Affiliations:** 1 Center for Reproductive Medicine, Cleveland Clinic, Cleveland, OH, USA

**Keywords:** Antioxidants, Assisted reproduction, Environmental pollutants, Female infertility, Lifestyle factors, Oxidative stress, Reactive oxygen species, Reproductive pathology

## Abstract

Oxidative stress (OS), a state characterized by an imbalance between pro-oxidant molecules including reactive oxygen and nitrogen species, and antioxidant defenses, has been identified to play a key role in the pathogenesis of subfertility in both males and females. The adverse effects of OS on sperm quality and functions have been well documented. In females, on the other hand, the impact of OS on oocytes and reproductive functions remains unclear. This imbalance between pro-oxidants and antioxidants can lead to a number of reproductive diseases such as endometriosis, polycystic ovary syndrome (PCOS), and unexplained infertility. Pregnancy complications such as spontaneous abortion, recurrent pregnancy loss, and preeclampsia, can also develop in response to OS. Studies have shown that extremes of body weight and lifestyle factors such as cigarette smoking, alcohol use, and recreational drug use can promote excess free radical production, which could affect fertility. Exposures to environmental pollutants are of increasing concern, as they too have been found to trigger oxidative states, possibly contributing to female infertility. This article will review the currently available literature on the roles of reactive species and OS in both normal and abnormal reproductive physiological processes. Antioxidant supplementation may be effective in controlling the production of ROS and continues to be explored as a potential strategy to overcome reproductive disorders associated with infertility. However, investigations conducted to date have been through animal or in vitro studies, which have produced largely conflicting results. The impact of OS on assisted reproductive techniques (ART) will be addressed, in addition to the possible benefits of antioxidant supplementation of ART culture media to increase the likelihood for ART success. Future randomized controlled clinical trials on humans are necessary to elucidate the precise mechanisms through which OS affects female reproductive abilities, and will facilitate further explorations of the possible benefits of antioxidants to treat infertility.

## Table of contents

1. Background

2. Reactive oxygen species and their physiological actions

3. Reactive nitrogen species

4. Antioxidant defense mechanisms

  4.1. Enzymatic antioxidants

  4.2. Non-enzymatic antioxidants

5. Mechanisms of redox cell signaling

6. Oxidative stress in male reproduction- a brief overview

7. Oxidative stress in female reproduction

8. Age-related fertility decline and menopause

9. Reproductive diseases

  9.1. Endometriosis

  9.2. Polycystic ovary syndrome

  9.3. Unexplained infertility

10. Pregnancy complications

  10.1. The placenta

  10.2. Spontaneous abortion

  10.3. Recurrent pregnancy loss

  10.4. Preeclampsia

  10.5. Intrauterine growth restriction

10.6. Preterm labor

11. Body weight

  11.1. Obesity/Overnutrition

  11.2. Malnutrition/Underweight

  11.3. Exercise

12. Lifestyle factors

12.1. Cigarette smoking

  12.2. Alcohol use

  12.3. Recreational drug use

   12.3.1. Cannabinoids

   12.3.2. Cocaine

13. Environmental and occupational exposures

  13.1. Organochlorine pesticides: DDT

  13.2. Polychlorinated biphenyls

  13.3. Organophosphate pesticides

14. Assisted reproductive techniques

15. Concluding remarks

16. Abbreviations

17. Competing interests

18. Authors’ contributions

19. Acknowledgements

20. References

## 1. Background

**Figure 1:**
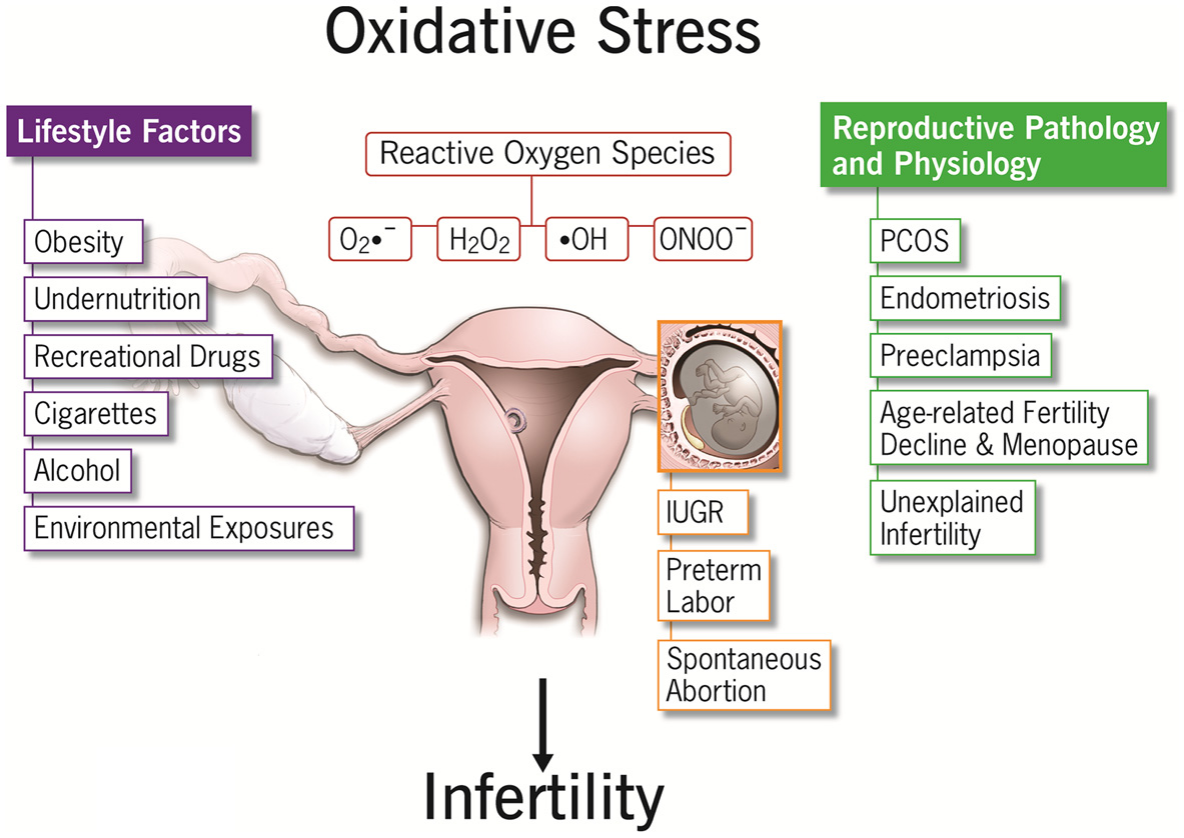
Factors contributing to the development of oxidative stress and their impacts on female reproduction.

Oxidative stress (OS) is caused by an imbalance between pro-oxidants and antioxidants [1]. This ratio can be altered by increased levels of reactive oxygen species (ROS) and/or reactive nitrogen species (RNS), or a decrease in antioxidant defense mechanisms [2-4]. A certain amount of ROS is needed for the progression of normal cell functions, provided that upon oxidation, every molecule returns to its reduced state [5]. Excessive ROS production, however, may overpower the body’s natural antioxidant defense system, creating an environment unsuitable for normal female physiological reactions [1] (Figure 1). This, in turn, can lead to a number of reproductive diseases including endometriosis, polycystic ovary syndrome (PCOS), and unexplained infertility. It can also cause complications during pregnancy, such spontaneous abortion, recurrent pregnancy loss (RPL), preeclampsia, and intrauterine growth restriction (IUGR) [6]. This article will review current literature regarding the role of ROS, RNS, and the effects of OS in normal and disturbed physiological processes in both the mother and fetus. The impact of maternal lifestyle factors exposure to environmental pollutants will also be addressed with regard to female subfertility and abnormal pregnancy outcomes. Obesity and malnutrition [4], along with controllable lifestyle choices such as smoking, alcohol, and recreational drug use [7] have been linked to oxidative disturbances. Environmental and occupational exposures to ovo-toxicants can also alter reproductive stability [8-10]. Infertile couples often turn to assisted reproductive techniques (ART) to improve their chances of conception. The role of supplementation of ART culture media with antioxidants continues to be of interest to increase the probability for ART success.

## 2. Reactive oxygen species and their physiological actions

Reactive oxygen species are generated during crucial processes of oxygen (O_2_) consumption [11]. They consist of free and non-free radical intermediates, with the former being the most reactive. This reactivity arises from one or more unpaired electrons in the atom’s outer shell. In addition, biological processes that depend on O_2_ and nitrogen have gained greater importance because their end-products are usually found in states of high metabolic requirements, such as pathological processes or external environmental interactions [2].

Biological systems contain an abundant amount of O_2_. As a diradical, O_2_ readily reacts rapidly with other radicals. Free radicals are often generated from O_2_ itself, and partially reduced species result from normal metabolic processes in the body. Reactive oxygen species are prominent and potentially toxic intermediates, which are commonly involved in OS [12].

The Haber-Weiss reaction, given below, is the major mechanism by which the highly reactive hydroxyl radical (OH^*^) is generated [13]. This reaction can generate more toxic radicals through interactions between the superoxide (SO) anion and hydrogen peroxide (H_2_O_2_) [12,13].

(1) $${\text{O}}_{2}^{+-}+{\text{H}}_{2}{\text{O}}_{2}>{\text{O}}_{2}+{\text{OH}}^{-}+\text{OH}*$$

However, this reaction was found to be thermodynamically unfavorable in biological systems.

The Fenton reaction, which consists of two reactions, involves the use of a metal ion catalyst in order to generate OH^*^, as shown below [12].

(2) $$\begin{array}{l} {\text{Fe}}^{3+}+{\text{O}}_{2}^{\cdot -}>{\text{Fe}}^{2+}+{\text{O}}_{2}\\ {\text{Fe}}^{2+}+{\text{H}}_{2}{\text{O}}_{2}>{\text{Fe}}^{3+}+{\text{OH}}^{-}+{\text{OH}}^{*} \end{array}$$

Certain metallic cations, such as copper (Cu) and iron (Fe^2+/3+^) may contribute significantly to the generation of ROS. On the other hand, metallic ion chelators, such as ethylenediamine tetra-acetic acid (EDTA), and transferrin can bind these metal cations, and thereby inhibit their ROS-producing reactivity [14].

Physiological processes that use O_2_ as a substrate, such as oxygenase reactions and electron transfer (ET) reactions, create large amounts of ROS, of which the SO anion is the most common [5]. Most ROS are produced when electrons leak from the mitochondrial respiratory chain, also referred to as the electron transport chain (ETC) [11]. Other sources of the SO anion include the short electron chain in the endoplasmic reticulum (ER), cytochrome P450, and the enzyme nicotinamide adenine dinucleotide phosphate (NADPH) oxidase, which generates substantial quantities --especially during early pregnancy-- and other oxido-reductases [2,11].

Mitochondria are central to metabolic activities in cells, so any disturbance in their functions can lead to profoundly altered generation of adenine triphosphate (ATP). Energy from ATP is essential for gamete functions. Although mitochondria are major sites of ROS production, excessive ROS can affect functions of the mitochondria in oocytes and embryos. This mitochondrial dysfunction may lead to arrest of cell division, triggered by OS [15,16]. A moderate increase in ROS levels can stimulate cell growth and proliferation, and allows for the normal physiological functions. Conversely, excessive ROS will cause cellular injury (e.g., damage to DNA, lipid membranes, and proteins).

The SO anion is detoxified by superoxide dismutase (SOD) enzymes, which convert it to H_2_O_2_. Catalase and glutathione peroxidase (GPx) further degrade the end-product to water (H_2_O). Although H_2_O_2_ is technically not a free radical, it is usually referred to as one due to its involvement in the generation and breakdown of free radicals. The antioxidant defense must counterbalance the ROS concentration, since an increase in the SO anion and H_2_O_2_ may generate a more toxic hydroxyl radical; OH^*^ modifies purines and pyrimidines, causing DNA strand breaks and DNA damage [17].

By maintaining tissue homeostasis and purging damaged cells, apoptosis plays a key role in normal development. Apoptosis results from overproduction of ROS, inhibition of ETC, decreased antioxidant defenses, and apoptosis-activating proteins, amongst others [18].

## 3. Reactive nitrogen species

Reactive nitrogen species include nitric oxide (NO) and nitrogen dioxide (NO_2_) in addition to non-reactive species such as peroxynitrite (ONOO^−^), and nitrosamines [19]. In mammals, RNS are mainly derived from NO, which is formed from O_2_ and L-arginine, and its reaction with the SO anion, which forms peroxynitrite [2]. Peroxynitrite is capable of inducing lipid peroxidation and nitrosation of many tyrosine molecules that normally act as mediators of enzyme function and signal transduction [19].

Nitric oxide is a free radical with vasodilatory properties and is an important cellular signaling molecule involved in many physiological and pathological processes. Although the vasodilatory effects of NO can be therapeutic, excessive production of RNS can affect protein structure and function, and thus, can cause changes in catalytic enzyme activity, alter cytoskeletal organization, and impair cell signal transduction [5,11]. Oxidative conditions disrupt vasomotor responses [20] and NO-related effects have also been proposed to occur through ROS production from the interaction between NO and the SO anion [21]. In the absence of L-arginine [19] and in sustained settings of low antioxidant status [20], the intracellular production of the SO anion increases. The elevation of the SO anion levels promotes reactions between itself and NO to generate peroxynitrite, which exacerbates cytotoxicity. As reviewed by Visioli et al (2011), the compromised bioavailability of NO is a key factor leading to the disruption of vascular functions related to infertile states [20]. Thus, cell survival is largely dependent on sustained physiological levels of NO [22].

Within a cell, the actions of NO are dependent on its levels, the redox status of the cell, and the amount of metals, proteins, and thiols, amongst other factors [19]. Since the effects of NO are concentration dependent, cyclic guanosine monophosphate (cGMP) has been thought to mediate NO-associated signal transduction as a second messenger at low (<1μ*M*) concentrations of NO [19,23].

The nitric oxide synthase (NOS) enzyme system catalyzes the formation of NO from O_2_ and L-arginine using NADPH as an electron donor [24] and are comprised of the following isoforms: neuronal NOS (nNOS or NOS I), inducible NOS (iNOS or NOS II), and endothelial NOS (eNOS or NOS III). In general, NO produced by eNOS and nNOS appears to regulate physiologic functions while iNOS production of NO is more active in pathophysiological situations. The NOS family is encoded by the genes for their isoforms. The nNOS isoform functions as a neurotransmitter and iNOS is expressed primarily in macrophages following induction by cytokines. The activity of eNOS is increased in response to the luteinizing hormone (LH) surge and human chorionic gonadotropin (hCG) [11].

The modulation of eNOS activity by increased intracellular calcium concentrations ([Ca^2+^_i_), which may occur acutely in response to agonists, including estradiol [25] and vascular endothelial growth factor (VEGF) [26]. However, the continued influx of Ca^2+^ across the plasma membrane that results in elevated [Ca^2+^_i_, is known as capacitative calcium entry (CCE), and is essential for maintaining eNOS activity [27] and regulating vascular tone [28,29]. In normal long-term conditions such as healthy pregnancies, vasodilation is particularly prominent in the uterine vessels [28,29]. During pregnancy, adaptation to sustained [Ca^2+^_i_ influx and elevation through the CCE response is imperative to eNOS activation [30-33] and is chiefly noted by vascular changes associated with normal pregnancy. Hypoxic conditions also regulate NOS [34] and enhanced expression of eNOS has been reported in ovine uterine arteries in response to chronic hypoxia [35]. Conversely, suboptimal vascular endothelial production of NO has been shown to cause hypertension not only in eNOS knockout mice [36,37], but more importantly, in humans [38]. Furthermore, failure of pregnancy states to adapt to sustained vasodilation [20] induced by the CCE signaling response can lead to complications such as IUGR [28] and preeclampsia, in which hypertension could be fatal [30].

## 4. Antioxidant defense mechanisms

Antioxidants are scavengers that detoxify excess ROS, which helps maintain the body’s delicate oxidant/antioxidant balance. There are two types of antioxidants: *enzymatic* and *non-enzymatic*.

### 4.1. Enzymatic antioxidants

Enzymatic antioxidants possess a metallic center, which gives them the ability to take on different valences as they transfer electrons to balance molecules for the detoxification process. They neutralize excess ROS and prevent damage to cell structures. Endogenous antioxidants enzymes include SOD, catalase, GPx, and glutathione oxidase.

Dismutation of the SO anion to H_2_O_2_ by SOD is fundamental to anti-oxidative reactions. The enzyme SOD exists as three isoenzymes [11]: SOD 1, SOD 2, and SOD 3. SOD 1 contains Cu and zinc (Zn) as metal co-factors and is located in the cytosol. SOD 2 is a mitochondrial isoform containing manganese (Mn), and SOD 3 encodes the extracellular form. SOD 3 is structurally similar to Cu,Zn-SOD, as it contains Cu and Zn as cofactors.

The glutathione (GSH) family of enzymes includes GPx, GST, and GSH reductase. GPx uses the reduced form of GSH as an H^+^ donor to degrade peroxides. Depletion of GSH results in DNA damage and increased H_2_O_2_ concentrations; as such, GSH is an essential antioxidant. During the reduction of H_2_O_2_ to H_2_Oand O_2_, GSH is oxidized to GSSG by GPx. Glutathione reductase participates in the reverse reaction, and utilizes the transfer of a donor proton from NADPH to GSSG, thus, recycling GSH [39].

Glutathione peroxidase exists as five isoforms in the body: GPx1, GPx2, GPx3, GPx4 [11], and GPx5 [39]. GPx1 is the cytosolic isoform that is widely distributed in tissues, while GPx2 encodes a gastrointestinal form with no specific function; GPx3 is present in plasma and epididymal fluid. GPx 4 specifically detoxifies phospholipid hydroperoxide within biological membranes. Vitamin E (α-tocopherol) protects GPx4-deficient cells from cell death [40]. GPx5 is found in the epididymis [39]. Glutathione is the major thiol buffer in cells, and is formed in the cytosol from cysteine, glutamate, and glycine. Its levels are regulated through its formation de-novo, which is catalyzed by the enzymes γ-glutamylcysteine synthetase and glutathione synthetase [4,11]. In cells, GSH plays multiple roles, which include the maintenance of cells in a reduced state and formation of conjugates with some hazardous endogenous and xenobiotic compounds.

### 4.2. Non-enzymatic antioxidants

The non-enzymatic antioxidants consist of dietary supplements and synthetic antioxidants such as vitamin C, GSH, taurine, hypotaurine, vitamin E, Zn, selenium (Se), beta-carotene, and carotene [41].

Vitamin C (ascorbic acid) is a known redox catalyst that can reduce and neutralize ROS. Its reduced form is maintained through reactions with GSH and can be catalyzed by protein disulfide isomerase and glutaredoxins.

Glutathione is a peptide found in most forms of aerobic life as it is made in the cytosol from cysteine, glutamate, and glycine [42]; it is also the major non-enzymatic antioxidant found in oocytes and embryos. Its antioxidant properties stem from the thiol group of its cysteine component, which is a reducing agent that allows it to be reversibly oxidized and reduced to its stable form [42]. Levels of GSH are regulated by its formation de-novo, which is catalyzed by the enzymes gamma-GCS and glutathione synthetase [4,11]. Glutathione participates in reactions, including the formation of glutathione disulfide, which is transformed back to GSH by glutathione reductase at the expense of NADPH [17].

Cysteine and cysteamine (CSH) increase the GSH content of the oocyte. Cysteamine also acts as a scavenger and is an antioxidant essential for the maintenance of high GSH levels. Furthermore, CSH can be converted to another antioxidant, hypotaurine [43,44].

The concentrations of many amino acids, including taurine, fluctuate considerably during folliculogenesis. Taurine and hypotaurine are scavengers that help maintain redox homeostasis in gametes. Both neutralize lipid peroxidation products, and hypotaurine further neutralizes hydroxyl radicals [44].

Like GSH, the Thioredoxin (Trx) system regulates gene functions and coordinates various enzyme activities. It detoxifies H_2_O_2_ and converts it to its reduced state via Trx reductase [45]. Normally, Trx is bound to apoptosis-regulating signal kinase (ASK) 1, rendering it inactive. However, when the thiol group of Trx is oxidized by the SO anion, ASK1 detaches from Trx and becomes active leading to enhanced apoptosis. ASK1 can also be activated by exposure to H_2_O_2_ or hypoxia-reoxygenation, and inhibited by vitamins C and E [2]. The Trx system also plays a role in female reproduction and fetal development by being involved in cell growth, differentiation, and death. Incorrect protein folding and formation of disulfide bonds can occur through H^+^ ion release from the thiol group of cysteine, leading to disordered protein function, aggregation, and apoptosis [2].

Vitamin E (α-tocopherol) is a lipid soluble vitamin with antioxidant activity. It consists of eight tocopherols and tocotrienols. It plays a major role in antioxidant activities because it reacts with lipid radicals produced during lipid peroxidation [42]. This reaction produces oxidized α-tocopheroxyl radicals that can be transformed back to the active reduced form by reacting with other antioxidants like ascorbate, retinol, or ubiquinol.

The hormone melatonin is an antioxidant that, unlike vitamins C and E and GSH, is produced by the human body. In contrast to other antioxidants, however, melatonin cannot undergo redox cycling; once it is oxidized, melatonin is unable to return to its reduced state because it forms stable end-products after the reaction occurs. Transferrin and ferritin, both iron-binding proteins, play a role in antioxidant defense by preventing the catalyzation of free radicals through chelation [46]. Nutrients such as Se, Cu, and Zn are required for the activity of some antioxidant enzymes, although they have no antioxidant action themselves.

Oxidative stress occurs when the production of ROS exceeds levels of antioxidants and can have damaging effects on both male and female reproductive abilities. However, it should be recalled that OS is also considered a normal physiological state, which is essential for many metabolic processes and biological systems to promote cell survival.

## 5. Mechanisms of redox cell signaling

Redox states of oocyte and embryo metabolism are heavily determined by ETs that lead to oxidation or reduction, and are thus termed redox reactions [18]. Significant sources of ROS in Graffian follicles include macrophages, neutrophils, and granulosa cells. During folliculogenesis, oocytes are protected from oxidative damage by antioxidants such as catalase, SOD, glutathione transferase, paraoxanase, heat shock protein (HSP) 27, and protein isomerase [47].

Once assembled, ROS are capable of reacting with other molecules to disrupt many cellular components and processes. The continuous production of ROS in excess can induce negative outcomes of many signaling processes [18]. Reactive oxygen species do not always directly target the pathway; instead, they may produce abnormal outcomes by acting as second messengers in some intermediary reactions [48].

Damage induced by ROS can occur through the modulation of cytokine expression and pro-inflammatory substrates via activation of redox-sensitive transcription factors AP-1, p53, and NF-kappa B. Under stable conditions, NF-kappa B remains inactive by inhibitory subunit I-kappa B. The increase of pro-inflammatory cytokines interleukin (IL) 1-beta and tumor necrosis factor (TNF)-alpha activates the apoptotic cascade, causing cell death. Conversely, the antioxidants vitamin C and E, and sulfalazine can prevent this damage by inhibiting the activation of NF-kappa B [3].

Deleterious attacks from excess ROS may ultimately end in cell death and necrosis. These harmful attacks are mediated by the following more specialized mechanisms [2].

A. *Opening of ion channels*: Excess ROS leads to the release of Ca^2+^ from the ER, resulting in mitochondrial permeability. Consequently, the mitochondrial membrane potential becomes unstable and ATP production ceases.

B. *Lipid peroxidation*: This occurs in areas where polyunsaturated fatty acid side chains are prevalent. These chains react with O_2_, creating the peroxyl radical, which can obtain H^+^ from another fatty acid, creating a continuous reaction. Vitamin E can break this chain reaction due to its lipid solubility and hydrophobic tail.

C.*Protein modifications*: Amino acids are targets for oxidative damage. Direct oxidation of side chains can lead to the formation of carbonyl groups.

D. *DNA oxidation*: Mitochondrial DNA is particularly prone to ROS attack due to the presence of O_2_^-^ in the ETC, lack of histone protection, and absence of repair mechanisms.

Reactive oxygen species are known to promote tyrosine phosphorylation by heightening the effects of tyrosine kinases and preventing those of tyrosine phosphatases. The inhibition of tyrosine phosphatases by ROS takes place at the cysteine residue of their active site. One possible mechanism of this inhibition is that it occurs through the addition of H_2_O_2_, which binds the cysteine residue and converts it to sulfenic acid. Another possible mechanism of inhibition is through the production of GSH via reduction from its oxidized form of GSSG; this conversion alters the catalytic cysteine residue site [49].

The human body is composed of many important signaling pathways. Amongst the most important signaling pathways in the body are the mitogen-activated protein kinases (MAPK). MAPK pathways are major regulators of gene transcription in response to OS. Their signaling cascades are controlled by phosphorylation and dephosphorylation of serine and/or threonine residues. This process promotes the actions of receptor tyrosine kinases, protein tyrosine kinases, receptors of cytokines, and growth factors [50,51]. Excessive amounts of ROS can disrupt the normal effects of these cascade-signaling pathways. Other pathways that can be activated by ROS include the c-Jun *N*-terminal kinases (JNK) and p38 pathways. The JNK pathway prevents phosphorylation due to its inhibition by the enzyme GST. The addition of H_2_O_2_ to this cascade can disrupt the complex and promote phosphorylation [52,53]. The presence of ROS can also dissociate the ASK1–Trx complex by activating the kinase [54] through the mechanism discussed earlier.

The concentration of Ca^2+^ must be tightly regulated as it plays an important role in many physiological processes. The presence of excessive amounts of ROS can increase Ca^2+^ levels, thereby promoting its involvement in pathways such as caldmodulin-dependent pathways [49,55]. Hypoxia-inducible factors (HIF) are controlled by O_2_ concentration. They are essential for normal embryonic growth and development. Low O_2_ levels can alter HIF regulatory processes by activating erythropoietin, another essential factor for proper embryonic growth and development [55,56].

The preservation of physiological cellular functions depends on the homeostatic balance between oxidants and antioxidants. Oxidative stress negatively alters cell-signaling mechanisms, thereby disrupting the physiologic processes required for cell growth and proliferation.

## 6. Oxidative stress in male reproduction- a brief overview

Almost half of infertility cases are caused by male reproductive pathologies [57], which can be congenital or acquired. Both types of pathology can impair spermatogenesis and fertility [58,59]. In males, the role of OS in pathologies has long been recognized as a significant contributor to infertility. Men with high OS levels or DNA damaged sperm are likely to be infertile [60].

The key predictors of fertilization capability are sperm count and motility. These essential factors can be disturbed by ROS [60] and much importance has been given to OS as a major contributor to infertility in males [61].

Low levels of ROS are necessary to optimize the maturation and function of spermatozoa. The main sources of seminal ROS are immature spermatozoa and leukocytes [4]. In addition, acrosome reactions, motility, sperm capacitation, and fusion of the sperm membrane and the oolemma are especially dependent on the presence of ROS [4,60].

On the other hand, inappropriately high levels of ROS produced by spermatozoa trigger lipid peroxidation, which damages the sperm’s plasma membrane and causes OS. Abnormal and non-viable spermatozoa can generate additional ROS and RNS, which can disrupt normal sperm development and maturation and may even result in apoptosis [4]. Specifically, H_2_O_2_ and the SO anion are perceived as main instigators of defective sperm functioning in infertile males [60]. Abnormally high seminal ROS production may alter sperm motility and morphology, thus impairing their capacity to fertilize [62].

The contribution of OS to male infertility has been well documented and extensively studied. On the other hand, the role of OS in female infertility continues to emerge as a topic of interest, and thus, the majority of conducted studies provide indirect and inconclusive evidence regarding the oxidative effects on female reproduction.

## 7. Oxidative stress in female reproduction

Each month, a cohort of oocytes begin to grow and develop in the ovary, but meiosis I resumes in only one of them, the dominant oocyte. This process is targeted by an increase in ROS and inhibited by antioxidants. In contrast, the progression of meiosis II is promoted by antioxidants [42], suggesting that there is a complex relationship between ROS and antioxidants in the ovary. The increase in steroid production in the growing follicle causes an increase in P450, resulting in ROS formation. Reactive oxygen species produced by the pre-ovulatory follicle are considered important inducers for ovulation [4]. Oxygen deprivation stimulates follicular angiogenesis, which is important for adequate growth and development of the ovarian follicle. Follicular ROS promotes apoptosis, whereas GSH and follicular stimulating hormone (FSH) counterbalance this action in the growing follicle. Estrogen increases in response to FSH, triggering the generation of catalase in the dominant follicle, and thus avoiding apoptosis [42].

Ovulation is essential for reproduction and commences by the LH surge, which promotes important physiological changes that result in the release of a mature ovum. An overabundance of post-LH surge inflammatory precursors generates ROS; on the other hand, depletion of these precursors impairs ovulation [46].

In the ovaries, the corpus luteum is produced after ovulation; it produces progesterone, which is indispensable for a successful pregnancy. Reactive oxygen species are also produced in the corpus luteum and are key factors for reproduction. When pregnancy does not occur, the corpus luteum regresses. Conversely, when pregnancy takes place, the corpus luteum persists [63]. A rapid decline in progesterone is needed for adequate follicle development in the next cycle. Cu,Zn-SOD increases in the corpus luteum during the early to mid-luteal phase and decreases during the regression phase. This activity parallels the change in progesterone concentration, in contrast to lipid peroxide levels, which increase during the regression phase. The decrease in Cu,Zn-SOD concentration could explain the increase in ROS concentration during regression. Other possible explanations for decreased Cu,Zn-SOD are an increase in prostaglandin (PG) F2-alpha or macrophages, or a decrease in ovarian blood flow [42]. Prostaglandin F2-alpha stimulates production of the SO anion by luteal cells and phagocytic leukocytes in the corpus luteum. Decreased ovarian blood flow causes tissue damage by ROS production. Concentrations of Mn-SOD in the corpus luteum during regression increase to scavenge the ROS produced in the mitochondria by inflammatory reactions and cytokines. Complete disruption of the corpus luteum causes a substantial decrease of Mn-SOD in the regressed cell. At this point, cell death is imminent [46]. The Cu,Zn-SOD enzyme is intimately related to progesterone production, while Mn-SOD protects luteal cells from OS-induced inflammation [42].

During normal pregnancy, leukocyte activation produces an inflammatory response, which is associated with increased production of SO anions in the 1^st^ trimester [64,65]. Importantly, OS during the 2^nd^ trimester of pregnancy is considered a normal occurrence, and is supported by mitochondrial production of lipid peroxides, free radicals, and vitamin E in the placenta that increases as gestation progresses [66-69].

## 8. Age-related fertility decline and menopause

Aging is defined as the gradual loss of organ and tissue functions. Oocyte quality decreases in relation to increasing maternal age. Recent studies have shown that low quality oocytes contain increased mtDNA damage and chromosomal aneuploidy, secondary to age-related dysfunctions. These mitochondrial changes may arise from excessive ROS, which occurs through the opening of ion channels (e.g. loss of Ca^2+^ homeostasis). Levels of 8-oxodeoxyguanosine (8-OHdG), an oxidized derivative of deoxyguanosine, are higher in aging oocytes. In fact, 8-OHdG is the most common base modification in mutagenic damage and is used as a biomarker of OS [70].

Oxidative stress, iron stores, blood lipids, and body fat typically increase with age, especially after menopause. The cessation of menses leads to an increase in iron levels throughout the body. Elevated iron stores could induce oxidative imbalance, which may explain why the incidence of heart disease is higher in postmenopausal than premenopausal women [71].

Menopause also leads to a decrease in estrogen and the loss of its protective effects against oxidative damage to the endometrium [72]. Hormone replacement therapy (HRT) may be beneficial against OS by antagonizing the effects of lower antioxidant levels that normally occurs with aging. However, further studies are necessary to determine if HRT can effectively improve age-related fertility decline.

## 9. Reproductive diseases

### 9.1. Endometriosis

Endometriosis is a benign, estrogen-dependent, chronic gynecological disorder characterized by the presence of endometrial tissue outside the uterus. Lesions are usually located on dependent surfaces in the pelvis and most often affect the ovaries and cul-de-sac. They can also be found in other areas such as the abdominal viscera, the lungs, and the urinary tract. Endometriosis affects 6% to 10% of women of reproductive age and is known to be associated with pelvic pain and infertility [73], although it is a complex and multifactorial disease that cannot be explained by a single theory, but by a combination of theories. These may include retrograde menstruation, impaired immunologic response, genetic predisposition, and inflammatory components [74]. The mechanism that most likely explains pelvic endometriosis is the theory of retrograde menstruation and implantation. This theory poses that the backflow of endometrial tissue through the fallopian tubes during menstruation explains its extra-tubal locations and adherence to the pelvic viscera [75].

Studies have reported mixed results regarding detection of OS markers in patients with endometriosis. While some studies failed to observe increased OS in the peritoneal fluid or circulation of patients with endometriosis [76-78], others have reported increased levels of OS markers in those with the disease [79-83]. The peritoneal fluid of patients have been found to contain high concentrations of malondialdehyde (MDA), pro-inflammatory cytokines (IL-6, TNF-alpha, and IL-beta), angiogenic factors (IL-8 and VEGF), monocyte chemoattractant protein-1 [82], and oxidized LDL (ox-LDL) [84]. Pro-inflammatory and chemotactic cytokines play a central role in the recruitment and activation of phagocytic cells, which are the main producers of both ROS and RNS [82].

Non-enzymatic peroxidation of arachidonic acid leads to the production of F2-isoprostanes [85]. Lipid peroxidation, and thus, OS in vivo [83], has been demonstrated by increased levels of the biomarker 8-iso-prostaglandin F2-alpha (8-iso-PGF2-alpha) [86-88]. Along with its vasoconstrictive properties, 8-iso-PGF2-alpha promotes necrosis of endothelial cells and their adhesion to monocytes and polymorphonuclear cells [89]. A study by Sharma et al (2010) measured peritoneal fluid and plasma levels of 8-iso-PGF2-alpha in vivo of patients with endometriosis. They found that 8-iso-PGF2-alpha levels in both the urine and peritoneal fluid of patients with endometriosis were significantly elevated when compared with those of controls [83]. Levels of 8-iso-PGF2-alpha are likely to be useful in predicting oxidative status in diseases such as endometriosis, and might be instrumental in determining the cause of concurrent infertility.

A collective term often used in reference to individual members of the HSP70 family is ‘HSP70’ [90]. The main inducible forms of HSP70 are HSPA1A and HSPA1B [91], also known as HSP70A and HSP70 B respectively [90]. Both forms have been reported as individual markers of different pathological processes [92].

Heat shock protein 70 B is an inducible member of HSP family that is present in low levels under normal conditions [93] and in high levels [94] under situations of stress. It functions as a chaperone for proteostatic processes such as folding and translocation, while maintaining quality control [95]. It has also been noted to promote cell proliferation through the suppression of apoptosis, especially when expressed in high levels, as noted in many tumor cells [94,96-98]. As such, HSP70 is overexpressed when there is an increased number of misfolded proteins, and thus, an overabundance of ROS [94]. The release of HSP70 during OS stimulates the expression of inflammatory cytokines [93,99] TNF-alpha, IL-1 beta, and IL-6, in macrophages through toll-like receptors (e.g. TLR 4), possibly accounting for pelvic inflammation and growth of endometriotic tissue [99].

Another inducible form of HSP70 known as HSP70b′ has recently become of great interest as it presents only during conditions of cellular stress [100]. Lambrinoudaki et al (2009) have reported high concentrations of HSP70b′ in the circulation of patients with endometriosis [101]. Elevated circulating levels of HSP70b′ may indicate the presence of OS outside the pelvic cavity when ectopic endometrial tissue is found in distal locations [101].

Fragmentation of HSP70 has been suggested to result in unregulated expression of transcription factor NF-kappa B [102], which may further promote inflammation within the pelvic cavity of patients with endometriosis. Oxidants have been proposed to encourage growth of ectopic endometrial tissue through the induction of cytokines and growth factors [103]. Signaling mediated by NF-kappa B stimulates inflammation, invasion, angiogenesis, and cell proliferation; it also prevents apoptosis of endometriotic cells. Activation of NF-kappa B by OS has been detected in endometriotic lesions and peritoneal macrophages of patients with endometriosis [104]. N-acetylcysteine (NAC) and vitamin E are antioxidants that limit the proliferation of endometriotic cells [105], likely by inhibiting activation of NF-kappa B [106]. Future studies may implicate a therapeutic effect of NAC and vitamin E supplementation on endometriotic growth.

Similar to tumor cells, endometriotic cells [107] have demonstrated increased ROS and subsequent cellular proliferation, which have been suggested to occur through activation of MAPK extracellular regulated kinase (ERK1/2) [108]. The survival of human endometriotic cells through the activation of MAPK ERK 1/2, NF-kappa B, and other pathways have also been attributed to PG E2, which acts through receptors EP2 and EP4 [109] to inhibit apoptosis [110]. This may explain the increased expressions of these proteins in ectopic versus eutopic endometrial tissue [109].

Iron mediates production of ROS via the Fenton reaction and induces OS [111]. In the peritoneum of patients with endometriosis, accumulation of iron and heme around endometriotic lesions [112] from retrograde menstruation [113] up-regulates iNOS activity and generation of NO by peritoneal macrophages [114]. Extensive degradation of DNA by iron and heme accounts for their considerable free radical activity. Chronic oxidative insults from iron buildup within endometriotic lesions may be a key factor in the development of the disease [115].

Naturally, endometriotic cysts contain high levels of free iron as a result of recurrent cyclical hemorrhage into them compared to other types of ovarian cysts. However, high concentrations of lipid peroxides, 8-OHdG, and antioxidant markers in endometrial cysts indicate lipid peroxidation, DNA damage, and up-regulated antioxidant defenses respectively. These findings strongly suggest altered redox status within endometrial cysts [111].

Potential therapies have been suggested to prevent iron-stimulated generation of ROS and DNA damage. Based on results from their studies of human endometrium, Kobayashi et al (2009) have proposed a role for iron chelators such as dexrazoxane, deferoxamine, and deferasirox to prevent the accumulation of iron in and around endometriotic lesions [115]. Future studies investigating the use of iron chelators may prove beneficial in the prevention of lesion formation and the reduction of lesion size.

Many genes encoding antioxidant enzymes and proteins are recruited to combat excessive ROS and to prevent cell damage. Amongst these are Trx and Trx reductase, which sense altered redox status and help maintain cell survival against ROS [116]. Total thiol levels, used to predict total antioxidant capacity (TAC), have been found to be decreased in women with pelvic endometriosis and may contribute to their status of OS [81,101]. Conversely, results from a more recent study failed to correlate antioxidant nutrients with total thiol levels [117].

Patients with endometriosis tend to have lower pregnancy rates than women without the disease. Low oocyte and embryo quality in addition to spermatotoxic peritoneal fluid may be mediated by ROS and contribute to the subfertility experienced by patients with endometriosis [118]. The peritoneal fluid of women with endometriosis contains low concentrations of the antioxidants ascorbic acid [82] and GPx [81]. The reduction in GPx levels was proposed to be secondary to decreased progesterone response of endometrial cells [119]. The link between gene expression for progesterone resistance and OS may facilitate a better understanding of the pathogenesis of endometriosis.

It has been suggested that diets lacking adequate amounts of antioxidants may predispose some women to endometriosis [120]. Studies have shown decreased levels of OS markers in people who consume antioxidant rich diets or take antioxidant supplements [121-124]. In certain populations, women with endometriosis have been observed to have a lower intake of vitamins A, C [125], E [125-127], Cu, and Zn [125] than fertile women without the disease [125-127]. Daily supplementation with vitamins C and E for 4 months was found to decrease levels of OS markers in these patients, and was attributed to the increased intake of these vitamins and their possible synergistic effects. Pregnancy rates, however, did not improve [126].

Intraperitoneal administration of melatonin, a potent scavenger of free radicals, has been shown to cause regression of endometriotic lesions [128-130] by reducing OS [129,130]. These findings, however, were observed in rodent models of endometriosis, which may not closely resemble the disease in humans.

It is evident that endometriotic cells contain high levels of ROS; however, their precise origins remain unclear. Impaired detoxification processes lead to excess ROS and OS, and may be involved in increased cellular proliferation and inhibition of apoptosis in endometriotic cells. Further studies investigating dietary and supplemental antioxidant intake within different populations are warranted to determine if antioxidant status and/or intake play a role in the development, progression, or regression of endometriosis.

### 9.2. Polycystic ovary syndrome

Polycystic ovary syndrome is the most common endocrine abnormality of reproductive-aged women and has a prevalence of approximately 18%. It is a disorder characterized by hyperandrogenism, ovulatory dysfunction, and polycystic ovaries [131]. Clinical manifestations of PCOS commonly include menstrual disorders, which range from amenorrhea to menorrhagia. Skin disorders are also very prevalent amongst these women. Additionally, 90% of women with PCOS are unable to conceive.

Insulin resistance may be central to the etiology of PCOS. Signs of insulin resistance such as hypertension, obesity, and central fat distribution are associated with other serious conditions, such as metabolic syndrome, nonalcoholic fatty liver [132], and sleep apnea. All of these conditions are risk factors for long-term metabolic sequelae, such as cardiovascular disease and diabetes [133]. Most importantly, waist circumference, independent of body mass index (BMI), is responsible for an increase in oxLDL [71]. Insulin resistance and/or compensatory hyperinsulinemia increase the availability of both circulating androgen and androgen production by the adrenal gland and ovary mainly by decreasing sex hormone binding globulin (SHBG) [134].

Polycystic ovary syndrome is also associated with decreased antioxidant concentrations, and is thus considered an oxidative state [135]. The decrease in mitochondrial O_2_ consumption and GSH levels along with increased ROS production explains the mitochondrial dysfunction in PCOS patients [136]. The mononuclear cells of women with PCOS are increased in this inflammatory state [137], which occurs more so from a heightened response to hyperglycemia and C-reactive protein (CRP). Physiological hyperglycemia generates increased levels of ROS from mononuclear cells, which then activate the release of TNF-alpha and increase inflammatory transcription factor NF-kappa B. As a result, concentrations of TNF-alpha, a known mediator of insulin resistance, are further increased. The resultant OS creates an inflammatory environment that further increases insulin resistance and contributes to hyperandrogenism [138].

Lifestyle modification is the cornerstone treatment for women with PCOS. This includes exercise and a balanced diet, with a focus on caloric restriction [139]. However, if lifestyle modifications do not suffice, a variety of options for medical therapy exist. Combined oral contraceptives are considered the primary treatment for menstrual disorders. Currently, there is no clear primary treatment for hirsutism, although it is known that combination therapies seem to produce better results [138].

### 9.3. Unexplained infertility

Unexplained infertility is defined as the inability to conceive after 12 months of unprotected intercourse in couples where known causes of infertility have been ruled out. It is thus considered a diagnosis of exclusion. Unexplained infertility affects 15% of couples in the United States. Its pathophysiology remains unclear, although the literature suggests a possible contribution by increased levels of ROS, especially shown by increased levels of the lipid peroxidation marker, MDA [140,141] in comparison to antioxidant concentration in the peritoneal cavity [142]. The increased amounts of ROS in these patients are suggestive of a reduction in antioxidant defenses, including GSH and vitamin E [76]. The low antioxidant status of the peritoneal fluid may be a determinant factor in the pathogenesis of idiopathic infertility.

N-acetyl cysteine is a powerful antioxidant with anti-apoptotic effects. It is known to preserve vascular integrity and to lower levels of homocysteine, an inducer of OS and apoptosis. Badaiwy et al (2006) conducted a randomized, controlled, study in which NAC was compared with clomiphene citrate as a cofactor for ovulation induction in women with unexplained infertility [143]. The study, however, concluded that NAC was ineffective in inducing ovulation in patients in these patients [143].

Folate is a B9 vitamin that is considered indispensable for reproduction. It plays a role in amino acid metabolism and the methylation of proteins, lipids, and nucleic acids. Acquired or hereditary folate deficiency contributes to homocysteine accumulation. Recently, Altmae et al (2010) established that the most important variation in folate metabolism in terms of impact is methyl-tetra-hydrofolate reductase (MTHFR) gene polymorphism 677C/T [144]. The MTHFR enzyme participates in the conversion of homocysteine to methionine, a precursor for the methylation of DNA, lipids, and proteins. Polymorphisms in folate-metabolizing pathways of genes may account for the unexplained infertility seen in these women, as it disrupts homocysteine levels and subsequently alters homeostatic status. Impaired folate metabolism disturbs endometrial maturation and results in poor oocyte quality [144].

More studies are clearly needed to explore the efficacy of antioxidant supplementation as a possible management approach for these patients.

## 10. Pregnancy complications

### 10.1. The placenta

The placenta is a vital organ of pregnancy that serves as a maternal-fetal connection through which nutrient, O_2_, and hormone exchanges occur. It also provides protection and immunity to the developing fetus. In humans, normal placentation begins with proper trophoblastic invasion of the maternal spiral arteries and is the key event that triggers the onset of these placental activities [6].

The placental vasculature undergoes changes to ensure optimal maternal vascular perfusion. Prior to the unplugging of the maternal spiral arteries by trophoblastic plugs, the state of low O_2_ tension in early pregnancy gives rise to normal, physiological hypoxia [145]. During this time, the syncytiotrophoblast is devoid of antioxidants, and thus, remains vulnerable to oxidative damage [146,147].

Between 10 and 12 weeks of gestation, the trophoblastic plugs are dislodged from the maternal spiral arteries, flooding the intervillous space with maternal blood. This event is accompanied by a sharp rise in O_2_ tension [148], marking the establishment of full maternal arterial circulation to the placenta associated with an increase in ROS, which leads to OS [68].

At physiological concentrations, ROS stimulate cell proliferation and gene expression [149]. Placental acclimation to increased O_2_ tension and OS at the end of the 1^st^ trimester up-regulates antioxidant gene expression and activity to protect fetal tissue against the deleterious effects of ROS during the critical phases of embryogenesis and organogenesis [2]. Amongst the recognized placental antioxidants are heme oxygenase (HO)-1 and -2, Cu,Zn-SOD, catalase, and GPx [150].

If maternal blood flow reaches the intervillous space prematurely, placental OS can ensue too early and cause deterioration of the syncytiotrophoblast. This may give rise to a variety of complications including miscarriage [148,151,152], recurrent pregnancy loss [153], and preeclampsia, amongst others [154]. These complications will be discussed below.

### 10.2. Spontaneous abortion

Spontaneous abortion refers to the unintentional termination of a pregnancy before fetal viability at 20 weeks of gestation or when fetal weight is < 500 g. Recent studies have shown that 8% to 20% of recognized clinical pregnancies end by spontaneous abortion before 20 weeks. The etiology consists mainly of chromosomal abnormalities, which account for approximately 50% of all miscarriages. Congenital anomalies and maternal factors such as uterine anomalies, infection, diseases, and idiopathic causes constitute the remaining causes [155].

Overwhelming placental OS has been proposed as a causative factor of spontaneous abortion. As mentioned earlier, placentas of normal pregnancies experience an oxidative burst between 10 and 12 weeks of gestation. This OS returns to baseline upon the surge of antioxidant activity, as placental cells gradually acclimate to the newly oxidative surroundings [148]. In cases of miscarriage, the onset of maternal intraplacental circulation occurs prematurely and sporadically between 8 and 9 weeks of pregnancy in comparison to normal continuous pregnancies [148,152]. In these placentas, high levels of HSP70, nitrotyrosine [151,152], and markers of apoptosis have been reported in the villi, suggesting oxidative damage to the trophoblast with subsequent termination of the pregnancy [2]. Antioxidant enzymes are unable to counter increases in ROS at this point, since their expression and activity increases with gestational age [148]. When OS develops too early in pregnancy it can impair placental development and/or enhance syncytiotrophoblastic degeneration, culminating in pregnancy loss [155].

The activity of serum prolidase, a biomarker of extracellular matrix and collagen turnover, has been observed to be decreased in patients with early pregnancy loss. Its levels were also shown to negatively correlate with increased OS, possibly accounting for the heightened placental vascular resistance and endothelial dysfunction secondary to decreased and dysregulated collagen turnover [156].

Decreased activity of serum paraoxonase/arylesterase --a major determinant of high-density lipoprotein (HDL) antioxidant status-- was noted in patients with early pregnancy loss. A negative correlation with lipid hydroperoxide was also observed in these patients, indicating their high susceptibility to lipid peroxidation [157].

Oxidative stress can also affect homeostasis in the ER. Persistence of endoplasmic OS can further sustain ER stress, eventually increasing decidual cell apoptosis and resulting in early pregnancy loss [158].

Decreased detoxification ability of GPx may occur in the setting of Se deficiency, which has been linked to both spontaneous abortion [159,160] and recurrent pregnancy loss [160].

Apoptosis of placental tissues may result from OS-induced inflammatory processes triggered by a variety of factors. Several etiologies may underlie improper initiation of maternal blood flow to the intervillous space; yet it may be through this mechanism by which both spontaneous and recurrent pregnancy loss occur.

Antioxidant supplementation has been investigated in the prevention of early pregnancy loss, with the idea of replacing depleted antioxidant stores to combat an overwhelmingly oxidative environment. However, a meta-analysis of relevant studies failed to report supporting evidence of beneficial effects of antioxidant supplementation [161].

### 10.3. Recurrent pregnancy loss

Recurrent pregnancy loss is defined as a history of ≥ 3 consecutive pregnancy losses, and has an incidence of 1% to 3%. In 50% of cases, causative factors can be identified. In the remaining 50%, however, no defined cause can be detected [162,163], although studies have pointed to a role of OS in the etiology of recurrent pregnancy loss [18,164].

It has been more recently suggested that the maternal uterine spiral arteries of normal pregnancies may involve uterine natural killer (NK) cells as a regulator of proper development and remodeling. Angiogenic factors are known to play key roles in the maintenance of proper spiral artery remodeling. Thus, the involvement of uterine NK cells in RPL has been supported by the early pregnancy findings of increased levels of angiogenic factors secreted by uterine NK cells [165], as well as increased in vivo and in vitro endothelial cell angiogenesis induced by uterine NK cells [166] in patients with RPL. Women experiencing RPL have also been noted to have increased endometrial NK cells, which were positively correlated to endometrial vessel density. Accordingly, it has been suggested that an increase of uterine NK cells increases pre-implantation angiogenesis, leading to precocious intra-placental maternal circulation, and consequently, significantly increased OS early in pregnancy [153].

The syncytiotrophoblastic deterioration and OS that occur as a result of abnormal placentation may explain the heightened sensitivity of syncytiotrophoblasts to OS during the 1^st^ trimester, and could contribute significantly to idiopathic RPL [154]. In keeping with this idea, plasma lipid peroxides and GSH have been observed in increased levels, in addition to decreased levels of vitamin E and β-carotene in patients with RPL [167]. Furthermore, markedly increased levels of GSH have also been found in the plasma of women with a history of RPL, indicating a response to augmented OS [168]. Another study showed significantly low levels of the antioxidant enzymes GPx, SOD, and catalase in patients with idiopathic RPL, in addition to increased MDA levels [169].

Polymorphisms of antioxidant enzymes have been associated with a higher risk of RPL [170-172]. The null genotype polymorphism of GST enzymes found in some RPL patients has been reported as a risk factor for RPL [18].

Antioxidant supplementation may be the answer to restoring antioxidant defenses and combating the effects of placental apoptosis and inflammatory responses associated with extensive OS. In addition to its well-known antioxidant properties, NAC is rich in sulphydryl groups. Its thiol properties give it the ability to increase intracellular concentrations of GSH or directly scavenge free radicals [173,174]. Furthermore, the fetal toxicity, death in utero, and IUGR, induced by lipopolysaccharides, might be prevented by the antioxidant properties of NAC [175]. Importantly, Amin et al (2008) demonstrated that the combination of NAC + folic acid was effective in improving pregnancy outcomes in patients with unexplained RPL [176]. By inhibiting the release of pro-inflammatory cytokines [177], endothelial apoptosis, and oxidative genotoxicity [178], via maintenance of intracellular GSH levels, NAC may well prove promising to suppress OS-induced reactions and processes responsible for the oxidative damage seen in complicated pregnancies.

### 10.4. Preeclampsia

Preeclampsia is a complex multisystem disorder that can affect previously normotensive women. It is a leading cause of maternal and fetal morbidity and mortality worldwide, occurring in 3% to 14% of pregnancies [179,180] . Preeclampsia clinically presents as a blood pressure reading > 140/90 mm Hg, taken on two separate occasions at least 6 hours apart along with proteinuria (≥ 0.3 g protein in a 24-hour urine specimen or persistent 1+ (30 mg/dL) protein on dipstick) after 20 weeks of gestation.

Preeclampsia can develop before (early onset) or after (late onset) 34 weeks of gestation. The major pathophysiologic disturbances are focal vasospasm and a porous vascular tree that transfers fluid from the intravascular to the extravascular space. The exact mechanism of vasospasm is unclear, but research has shown that interactions between vasodilators and vasoconstrictors, such as NO, endothelin 1, angiotensin II, prostacyclin, and thromboxane, can cause decrease the perfusion of certain organs. The porous vascular tree is one of decreased colloid osmotic pressure and increased vascular permeability [181-183].

Placental ischemia/hypoxia is considered to play an important role through the induction of OS, which can lead to endothelial cell dysfunction [68,180] and systemic vasoconstriction [184]. From early pregnancy on, the body assumes a state of OS. Oxidative stress is important for normal physiological functions and for placental development [185]. Preeclampsia, however, represents a much higher state of OS than normal pregnancies do [186].

Early-onset preeclampsia is associated with elevated levels of protein carbonyls, lipid peroxides, nitrotyrosine residues, and DNA oxidation, which are all indicators of placental OS [68,187]. The OS of preeclampsia is thought to originate from insufficient spiral artery conversion [150,188,189] which leads to discontinuous placental perfusion and a low-level ischemia-reperfusion injury [185,190,191]. Ischemia-reperfusion injury stimulates trophoblastic and endothelial cell production of ROS [192], along with variations in gene expression that are similar to those seen in preeclampsia [3]. Oxidative stress can cause increased nitration of p38 MAPK, resulting in a reduction of its catalytic activity. This may cause the poor implantation and growth restriction observed in preeclampsia [6]. Exaggerated apoptosis of villous trophoblasts has been identified in patients with preeclampsia, of which OS has been suggested as a possible contributor. Microparticles of syncytiotrophoblast microvillus membrane (STBMs) have been found throughout the maternal circulation of patients with preeclampsia and are known to cause endothelial cell injury in vitro [193].

Placental OS can be detected through increased serum concentrations of ROS such as H_2_O_2_[194], or lipid peroxidation markers [195] such as MDA [179,195-197] and thiobarbituric acid reactive substances (TBARS) [179,194]. Increased circulating levels of the vasoconstrictor H_2_O_2_[188,194] and decreased levels of the vasodilator NO [194,198] have been noted in preeclampsia and may account for the vasoconstriction and hypertension present in the disease. Still, some studies have conversely reported increased circulating [199,200] and placental [201] NO levels. Neutrophil modulation occurring in preeclampsia is another important source of ROS, and results in increased production of the SO anion and decreased NO release, which ultimately cause endothelial cell damage in patients with preeclampsia [202].

The activation of ASK1, induced by H_2_O_2_ or hypoxia/reoxygenation, leads to elevated levels of soluble receptor for VEGF (sFlt-1) [203], which has anti-angiogenic properties [150,204]. Elevated circulating levels of sFlt-1 have been suggested to play a role in the pathogenesis of preeclampsia [203,204] and the associated endothelial dysfunction [204]. Placental trophoblastic hypoxia resulting in OS has been linked to excess sFlt-1 levels in the circulation of preeclamptic women [150]. Vitamins C and E, and sulfasalazine can decrease sFlt-1 levels [203].

Heme oxygenase-1 [205] is an antioxidant enzyme that has anti-inflammatory and cytoprotective properties. Hypoxia stimulates the expression of HO-1 [206] in cultured trophoblastic cells, and is used to detect increased OS therein [207]. Preeclampsia may be associated with decreased levels of HO in the placenta [205], suggesting a decline in protective mechanisms in the disease. More recently, decreased cellular mRNA expressions of HO-1, HO-2, SOD, GPx, and catalase were reported in the blood of preeclamptic patients [150,179,194]. Tissue from chorionic villous sampling of pregnant women who were diagnosed with preeclampsia later in gestation revealed considerably decreased expressions of HO-1 and SOD [208]. Failure to neutralize overwhelming OS may result in diminished antioxidant defenses.

Members of the family of NAD(P)H oxidases are important generators of the SO anion in many cells, including trophoblasts and vascular endothelial cells. Increased SO anion production through activation of these enzymes may occur through one of several physiological mechanisms, and has been implicated in the pathogenesis of some vascular diseases [209]. Autoantibodies against the angiotensin receptor AT1, particularly the second loop (AT1-AA) [210], can stimulate NAD(P)H oxidase, leading to increased generation of ROS. In cultured trophoblast and smooth muscle cells, the AT1 receptor of preeclamptic women has been observed to promote both the generation of the SO anion and overexpression of NAD(P)H oxidase [211]. Between 6 and 8 weeks of gestation, active placental NAD(P)H yields significantly more SO anion than is produced during full-term [212]. Thus, early placental development may be affected through dysregulated vascular development and function secondary to NAD(P)H oxidase-mediated altered gene expression [48,213]. Preeclamptic women produce ROS and exhibit higher NAD(P)H expression than those without the disease [211]. More specifically, it has been reported that women with early-onset preeclampsia produce higher amounts of the SO anion than women with late-onset disease [212]. Levels of TNF-α, and oxLDL are increased in preeclampsia and have been shown to activate the endothelial isoform of NAD(P)H oxidase been, ultimately resulting in increased levels of the SO anion [209]. The mechanism of placental NAD(P)H activation is still unclear, but the above findings may assist in elucidating the role of OS in the pathogenesis of placental dysfunction in reproductive diseases such as preeclampsia.

Paraoxonase-1 (PON 1), an enzyme associated with HDL, acts to offset LDL oxidation and prevent lipid peroxidation [214] in maternal serum. Baker et al (2010) demonstrated that PON 1 levels tend to be high in patients with preeclampsia, which suggests that OS contributes to the pathogenesis of the disease [215]. Paraoxonase-1 has also been measured to be increased in patients in mid-gestation [215], possibly in an attempt to shield against the toxic effects of high OS encountered in preeclampsia. In contrast, other studies have observed considerably decreased PON 1 in the presence of clinical symptoms [216,217] and in patients with severe preeclampsia [216]. These results indicate consumption of antioxidants to combat heightened lipid peroxidation, which may injure vascular endothelium, and likely be involved in the pathogenesis of preeclampsia [216,217].

Affected women also have a decreased total antioxidant status (TAS), placental GPx [179,195,218], and low levels of vitamins C and E [194]. Inadequate vitamin C intake seems to be associated with an increased risk of preeclampsia [219] and some studies have shown that peri-conceptional supplementation with multivitamins may lower the risk of preeclampsia in normal or under-weight women [220,221]. However, the majority of trials to date have found routine antioxidant supplementation during pregnancy to be ineffective in reducing the risk of preeclampsia [161,222-224].

### 10.5. Intrauterine growth restriction

Intra uterine growth restriction is defined as infant birth weight below the 10^th^ percentile. This condition affects 10% of newborns [225] and increases the risk for perinatal morbidity and mortality. Placental, maternal, and fetal factors are the most common causes of IUGR. Preeclampsia is an important cause of IUGR, as it develops from uteroplacental insufficiency and ischemic mechanisms in the placenta [226]. Studies also indicate that patients with IUGR develop OS because of placental ischemia/reperfusion injury secondary to improper spiral arteriole development. Imbalanced injury and repair as well as abnormal development of the villous tree are characteristic of IUGR placentas, predisposing them to depletion of the syncytiotrophoblast with consequently limited regulation of transport and secretory function. As such, OS is recognized as an important player in the development of IUGR [227].

Women with IUGR have been reported to have increased free radical activity and markers of lipid peroxidation [228]. Furthermore, Biri et al (2007) reported that higher levels of MDA and xanthine oxidase and lower levels of antioxidant concentrations in the plasma, placenta, and umbilical cords in patients with IUGR compared to controls [227]. Urinary *8-oxo-7,8- dihydro-2-deoxyguanosine* (8-OxOdG), a marker of DNA oxidation, was also observed to be elevated at 12 and 28 weeks in pregnancies complicated with growth-restricted fetuses compared with a control group [229].

Ischemia and reperfusion injury are powerful generators of ROS and OS. The regulatory apoptotic activity of p53 [227] is significantly increased in response to hypoxic conditions within villous trophoblasts [230-232] and signifies a greater degree of apoptosis secondary to hypoxia-reoxygenation [233] than from hypoxia alone [230]. Decreases in the translation and signaling of proteins add to the overwhelming OS in IUGR placentas [234].

Furthermore, disordered protein translation and signaling in the placenta can also cause ER stress in the syncytiotrophoblast, and has been demonstrated in placentas of IUGR patients [187]. ER stress inhibits placental protein synthesis, eventually triggering apoptosis [234]. Moreover, induction of p38 and NF-kappa B pathways can occur through ER stress, exacerbating inflammatory responses [187]. Disrupted Ca^2+^ homeostasis can lead to compromised perfusion and result in ER stress. The chronicity these events may explain the placental growth restriction seen in these pregnancies [235]. In addition, serum prolidase activity in patients with IUGR was significantly elevated and negatively correlated with TAC, suggesting increased and dysregulated collagen turnover [236].

The origin of these placental insults induced by OS and ER stress is not completely understood, but ischemia/reperfusion and hypoxia-reoxygenation are considered as significant contributors.

### 10.6. Preterm labor

Preterm labor occurs before 37 weeks of gestation and is the leading cause of perinatal morbidity and mortality worldwide with an incidence between 5% and 12%. Beyond their differences in timing, term and preterm labor have long been thought of as similar processes that occur through a ‘common pathway’. Although the precise etiologies and initiating mechanism of preterm labor remain unclear, the term “syndrome” has been used by Romero et al (2006) to describe possible pathological etiologies for the onset of premature labor [237].

The sequence of uterine contraction, cervical dilatation, and decidual activation make up the uterine component of this pathway [237]. However, it has been proposed that activation of this common pathway through physiological signals results in term labor, while preterm labor might occur from spontaneous activation of isolated aspects of the common pathway by the presence of pathological conditions that may be induced by multiple causes [238] or risk factors.

Preterm labor in general is divided in two distinctive types: *indicated*, usually due to maternal or fetal reasons, or *spontaneous*. The majority of spontaneous preterm deliveries occur from any of the four primary pathogenic pathways. These include uterine overdistension, ischemia, infection, cervical disease, endocrine disorders [237], decidual hemorrhage, and maternal-fetal activation of the hypothalamic-pituitary axis, amongst others [239]. Of these etiologies, intrauterine infection and inflammation is considered a main contributor to preterm birth [240].These pathogenic mechanisms converge on a common pathway involving increased protease expression and uterotonin. More than one process may take place in a given woman. The combination of genetics and inflammatory responses is an active area of research that could explain preterm labor in some women with common risk factors [241,242].

Labor induces changes in chorioamniotic membranes that are consistent with localized acute inflammatory responses, despite the absence of histological evidence of inflammation [243]. Reactive oxygen species activates NF-kappa B, which stimulates COX-2 expression and promotes inflammation with subsequent parturition. A study by Khan et al (2010) reported markedly decreased GPx protein expression in both women with preterm labor and those with term labor, compared with the respective non-labor groups [244]. Taken together, these data suggest that the state of labor, whether preterm or term, necessitates the actions of GPx to limit lipid oxidation, and is associated with an ROS-induced reduction of antioxidant defenses.

Mustafa et al (2010) detected markedly higher levels of MDA and 8-OHdG and significantly lower GSH levels in the maternal blood of women with preterm labor than in women with term deliveries [245]. This finding suggested that women in preterm labor have diminished antioxidant abilities to defend against OS-induced damage. Moreover, reduced activities of FRAP, an assay that measures a person’s ability to defend against to oxidative damage, and GST, have also been found in women with preterm labor [245-248]. The results further support that a maternal environment of increased OS and decreased antioxidants renders both the mother and fetus more susceptible to ROS-induced damage.

Inflammation induces the up-regulation of ROS and can cause overt OS, resulting in tissue injury and subsequent preterm labor [249]. The concentration of Mn-SOD increases as a protective response to inflammation and OS, and down-regulates NF-kappa B, activator protein-1, and MAPK pathways [250]. Accordingly, higher mRNA expression of Mn-SOD was observed in the fetal membranes of women in preterm labor than in women in spontaneous labor at term, which may suggest a greater extent of OS and inflammatory processes in the former [251].

Preterm labor has been associated with chorioamnionitis and histological infection was found to relate to elevated fetal membrane expression of Mn-SOD mRNA of women in preterm labor [251]. The increased Mn-SOD mRNA expressions in these cases may be a compensatory response to the presence of increased OS and inflammation in preterm labor.

Specifically, significantly higher amounts of the pro-inflammatory cytokines IL-1 beta, IL-6, and IL-8, have been observed in the amnion and choriodecidua of patients in preterm labor than in women in spontaneous term labor. These findings support activation of the membrane inflammatory response of women in preterm labor [252].

Women with preterm labor have lower levels of TAS than women with uncomplicated pregnancies at a similar gestational age, which might indicate the presence of increased OS during preterm labor [253]. Women with preterm births have also been found to have significantly decreased PON 1 activity in comparison to controls [254]. This finding suggests that enhanced lipid peroxidation and diminished antioxidant activity of PON 1, may together create a pro-oxidant setting and increase the risk for preterm birth. Additionally, patients in preterm labor had markedly decreased levels of GSH [255]. Low maternal serum selenium levels in early gestation have been associated with preterm birth [256]. Polymorphism to GST was found to be significantly higher in patients in preterm labor, indicating that these patients are more vulnerable to oxidative damage [245]. The inflammatory setting of maternal infection associated with preterm birth produces a state of OS and the consequent decrease in antioxidant defenses are likely to increase the risk for preterm birth.

The presented evidence implicates inflammation and suppressed antioxidant defenses in the pathogenesis of preterm labor. Thus, it seems plausible that antioxidant supplementation may assist in preventing preterm labor and birth associated with inflammation. A study by Temma-Asano et al (2011) demonstrated that NAC was effective in reducing chorioamnionitis-induced OS, and thus, may protect against preterm labor [257]. However, maternal supplementation with vitamins C and E in low-risk nulliparous patients during early gestation did not reduce preterm births [258,259]. Due to the conflicting results of studies, it is unclear whether maternal antioxidant supplementation plays a role in preventing the onset of preterm labor.

## 11. Body weight

Pregnancy is a state of increased metabolic demands required to support both maternal hormonal physiology and normal fetal development. However, inadequate or excessive pregnancy weight gain can complicate both maternal and fetal health [260]. The adverse effects of maternal obesity and underweight on fertility from disordered hormones and menses have been well-documented [260]. Ideally, women with a normal pre-pregnancy BMI (19.8-24.9) should gain between 25 and 35 pounds during pregnancy. Overweight women (BMI 25-29.9) should aim to gain between 15 and 25 pounds, and obese women (BMI >30) should gain no more than 15 pounds [261].

### 11.1. Obesity/overnutrition

Close to two-thirds of the United States population of reproductive-aged women are considered overweight or obese [226]. Obese women generally take longer to conceive and have a higher risk of miscarriage than their leaner counterparts [262]. Maternal obesity has also long been associated with several reproductive pathologies including gestational diabetes mellitus, preeclampsia, and PCOS. It has also been shown to negatively affect fertility and pregnancy. and Delivery complications and fetal complications such as macrosomia have also been linked to maternal obesity [263].

Healthy pregnancies are associated with the mobilization of lipids, increased lipid peroxides, insulin resistance, and enhanced endothelial function. Normally, increases in total body fat peak during the 2^nd^ trimester. Obese women, however, experience inappropriately increased lipid peroxide levels and limited progression of endothelial function during their pregnancies, along with an additive innate tendency for central fat storage. Visceral fat is associated with disordered metabolism and adipokine status, along with insulin resistance. Centrally-stored fat deposits are prone to fatty acid overflow, thereby exerting lipotoxic effects on female reproductive ability [264].

Oxidative stress from excessive ROS generation has been implicated in pathogenesis of obesity [265]. Intracellular fat accumulation can disrupt mitochondrial function, causing buildup and subsequent leak of electrons from the ETC. The combined effect of high lipid levels and OS stimulates production of oxidized lipids; of particular importance are lipid peroxides, oxidized lipoproteins, and oxysterols. As major energy producers for cells, the mitochondria synthesize ATP via oxidative phosphorylation. Adverse effects of maternal BMI on mitochondria in the oocyte could negatively influence embryonic metabolism.

Increased plasma non-esterified fatty acid levels can prompt the formation of the nitroxide radical. As a known inflammatory mediator, oxLDL can indirectly measure lipid-induced OS, hence elucidating its role in the inflammatory state of obesity [266]_._ Oxysterol production within a lipotoxic environment can potentially disrupt the placental development and function of obese pregnancies [267]. Consumption of a high fat meal has been shown to increase levels of both circulating endotoxins and markers of endothelial dysfunction [267-269].

Extensive evidence has linked endothelial dysfunction, increased vascular endothelial cell expression of NADPH oxidase, and endothelial OS to obesity. Overactive mitochondria and harmful ROS levels in oocytes and zygotes were influenced by peri-conceptional maternal obesity. Igosheva et al (2010) reported a decline in fertility and obscured progression of the developing embryo [264]. The correlation between placental nitrative stress from altered vascular endothelial NO release and high maternal BMI [270] may stem from imbalances of oxidative and nitrative stress, which may weaken protection to the placenta [271]. Results from Ruder et al (2009) supported the association of increased maternal body weight and increased nitrative stress, but did not demonstrate a relation to placental OS [4].

Overabundant nutrition may produce an unfavorably rich reproductive environment, leading to modified oocyte metabolism and hindered embryo development. A negative association was also made between maternal diet-induced obesity and blastocyst development [264]. Increased postprandial levels of OS biomarkers have been described after ingestion of high fat meals. A study by Bloomer et al (2009) found a greater increase in postprandial MDA in obese females versus normal weight controls [265]. Hallmark events of obese states include decreased fatty acid uptake, enhanced lipolysis, infiltration of inflammatory cells, and secretion of adipokines [267,272].

Suboptimal oocyte quality has also been noted in obese females. More specifically, follicular fluid (FF) levels of CRP were observed to be abnormally high [273]. The resultant disturbance of oocyte development may influence oocyte quality and perhaps general ovarian function.

Maternal obesity has been linked to several increased risks to the mother, embryo, and fetus. Obesity is considered a modifiable risk factor; therefore, pre-conceptional counseling should stress the importance of a balanced diet and gestational weight gain within normal limits.

### 11.2. Malnutrition/underweight

Nutritional deficiencies in underdeveloped areas of the world continue to be a significant public health concern. Inadequate maternal nutrition during the embryonic period adversely affects fetal growth, placing a pregnant woman at risk for a low birth weight infant and potential endothelial dysfunction.

Malnourished females and those with a low BMI may be at increased risk for impaired endothelium-dependent vasodilation secondary to OS [271]. In-utero undernutrition reduces NO stores, triggering OS along with impairment of endothelium-dependent vasodilation. In rodents, gestational exposure to both caloric and protein restriction resulted in low birth weight offspring. The activity of SOD was found to be decreased with a consequent increase of the SO anion in the offspring of undernourished dams, which also indicates decreased formation of H_2_O_2_. Elevated SO anion levels also stimulate NO scavenging and cell damage associated with endothelial dysfunction [274].

Concentrations of 8-OHdG and MDA commonly mark OS and are strikingly elevated in both low BMI and obese women in comparison to those with normal BMI. In particular, 8-OHdG is produced by hydroxyl radical interaction with DNA, and is valuable for the detection of oxidative DNA damage [271].

Primordial, secondary, and antral follicle numbers markedly decrease in relation to time intervals of limited nutritional exposure. Insufficient maternal nutrition, especially during critical periods of embryonic and fetal development, manifests as an overall elevation of ovarian OS, which, along with impaired mitochondrial antioxidant defenses, may be responsible for these significantly decreased follicle numbers and resultant growth impediment of offspring [275].

In general, adolescence is a period of increased physiological demands for growth and development. If a pregnancy occurs during this time, it creates an environment in which mother and fetus compete for nutrients, as both parties are undergoing major developmental changes throughout gestation. Inadequate nutrition during adolescence is especially problematic, as youths often lack one or more vital micronutrients. Given the varied requirements of different communities and populations for health maintenance, antioxidant or mineral supplementation should be population-specific.

### 11.2. Exercise

Physical exercise produces an oxidative state due to excessive ROS generation. Any type of extreme aerobic or anaerobic activity (e.g. marathon running, weight training) may contribute to cellular damage. Optimal amounts of OS are necessary for physiologic functioning. Physical activity causes an increase in ROS, which in turn heightens antioxidant response, thus providing protection from future attacks [276]. An overproduction of OS after acute exercise in certain diseased individuals may serve as a trigger for improved antioxidant defense when compared with their healthy counterparts [277]. Leelarungrayub et al (2010) established that aerobic exercise can increase TAC and decrease MDA levels, resulting in better physical fitness in previously sedentary women [278]. Maternal BMI has great potential to affect pregnancy outcomes and would likely benefit from further research.

## 12. Lifestyle factors

The 21st century has been burdened with a sharp increase in the use of several substances of abuse. This problem significantly affects the younger generations, which encompass the female reproductive years. Cigarette smoking, alcohol use, and recreational drug use have been implicated in the pathogenesis of perturbed female reproductive mechanisms, leading to increased times to conception and infertility [279].

### 12.1. Cigarette smoking

The nicotine component of cigarette smoke is notoriously addictive and toxic to the human body. In the United States, approximately one-third of women in the reproductive age group smoke cigarettes. Maternal smoking is associated with infertility, pregnancy complications, and damage to the developing embryo. Higher rates of fetal loss, decreased fetal growth [280], and preterm birth have also been associated with maternal smoking. The risk of spontaneous abortion has been found to be greatly increased in smokers versus non-smokers [281]. Many authors have proposed that nicotine receptors play a role in the aforementioned pathologies, but the influence of OS has only recently become of interest [7]. Evidence suggests that maternal cigarette smoking leads to OS in both mother and fetus [7,282].

Cigarette smoke is composed of many toxic chemicals and pro-oxidants that can produce ROS. The inhaled tobacco smoke is composed of two phases: the *particulate* (tar) phase containing stable free radicals, and the *gas* phase, which contains toxins and free radicals. Reactive oxygen species such as the SO anion, H_2_O_2_, and the hydroxyl radical are formed by water-soluble constituents of tar, and can damage fundamental parts of cells and DNA. Even exposure to passive smoke had been linked to decreased pregnancy rates and increased time to conception [273,282]. The harmful and carcinogenic effects of both smoke types have been well documented, and in general, no level of smoke exposure can be considered safe [283].

The principal components believed to be responsible for toxicity are nicotine and benzo[alpha]pyrene through high ROS formation and subsequent OS on the embryo and fetus [284,285]. In addition, a high free radical state can deplete protective antioxidants [286], namely vitamin E, beta-carotene, SOD, and catalase [7]. The impact of ROS and OS is thought to fluctuate with varying amounts of active smoke exposure [282]. Levels of TBARS have been observed in the plasma and tissues of smokers and correlate with the number of cigarettes smoked [287].

Nicotine iminium and myosamine iminium are the chief metabolites produced by oxidation of nicotine. The reduction potentials of these metabolites seem to permit in vivo ET and resultant OS [7].

NO is just one species contained in the gas phase. Overproduction of NO causes subsequent formation of peroxynitrite. Cigarette tar content positively correlates with the production of hydroxyl radical, a notorious inducer of DNA damage [283].

Increased risks of infertility, miscarriage, IUGR, and low birth weight have been extensively reported amongst pregnant smokers. A 12-study meta-analysis reported that smokers had a significantly increased odds ratio for infertility in addition to lengthened time to conception, both likely through the activation of OS mechanisms [4].

Further, delayed conception has been recorded in women undergoing in vitro fertilization (IVF) [282]. A recent meta-analysis of 21 studies also reported a significant decrease in the odds for pregnancy and live delivery per cycle versus non-smokers, as well as a marked increase in the odds for spontaneous miscarriage and ectopic pregnancy. In other ART studies, a decrease in fertilization rate was observed in smokers [288].

Cigarette smoke is a significant source of exogenous OS targeting the follicular microenvironment [288]. Smoking has been found to decrease FF β-carotene levels [4]. Tiboni et al (2004) found a sequestration of intrafollicular tobacco metabolites relating to cigarette smoke exposure. They also reported an additional association of cigarette smoke exposure to markedly increased follicular lipid peroxidation with parallel reduction of local antioxidant capacity. The study concluded that beta-carotene may be depleted as a result of consumed antioxidant defenses in response to smoke-induced ROS [288].

Chelchowska et al (2011) demonstrated decreased plasma vitamin A and beta-carotene concentrations in smokers compared to non-smokers [286]. They concluded that smoking during pregnancy stimulated a higher degree of lipid peroxidation than normal pregnancy. Similar findings were also observed in those exposed to passive smoke, suggesting that even those exposed to second-hand smoke may be subject to similar toxic effects as those who actively smoke [286].

Although normal pregnancy is associated with increased lipid peroxidation, conflicting data exists regarding MDA concentrations in pregnant female smokers. The additional free radical load from tobacco smoke causes an imbalance between oxidants and antioxidants. Results from Chelchowska et al (2011) positively correlated MDA concentrations with levels of cotinine-- a marker of tobacco smoke exposure-- in maternal smokers; additionally, a decreased antioxidant supply was also observed in smokers [286].

A study examining mouse oocytes reported decreased oocyte quality in association with cigarette smoke exposure. Embryos of mothers exposed to cigarette smoke showed defective development due to oxidative damage and cell death, possibly secondary to arrested cell cycles [280].

Several studies have demonstrated direct adverse effects of tobacco smoke on embryos and fetuses. Placental transfer of nicotine and carbon monoxide in tobacco smoke can induce placental hypoxia, leading to utero-placental insufficiency and inadequate delivery of O_2_ and nutrients to the developing fetus [282]. However, the effect of tobacco smoke on female fertility may be transient, exerting toxic effects during active maternal smoking, which reverse on smoking cessation [280].

### 12.2. Alcohol use

Even moderate alcohol use during pregnancy can result in IUGR and low birth weight, and increase the risk for congenital anomalies. Early pregnancy loss and spontaneous abortion are also strongly attributed to fetal exposure of maternal alcohol use [282].

Primary elimination of ethanol (EtOH) occurs through an oxidative mechanism via hepatic metabolism [289]. Upon ingestion, alcohol undergoes dehydrogenation to acetaldehyde [7,290]. Subsequent further dehydrogenation of acetaldehyde produces acetic acid with acetyl and methyl radicals. These metabolites are responsible for ROS generation. Regular alcohol use thus leads to overproduction of ROS, triggering lipid peroxidation, and lowering SOD antioxidant activity and reducing GSH levels. This toxicity is considered to be primarily inflicted by acetaldehyde, and possibly propagates redox cycling and catalytic generation of OS [7].

In a study by Gauthier et al (2010), maternal alcohol consumption of more than three drinks per occasion was found to produce prominent systemic OS. Postpartum subjects demonstrated a marked reduction of systemic GSH, along with significant increases in the percentage of oxidized GSSG and oxidation of the GSH redox potential [291].

The OS likely induced by EtOH metabolism [292,293] may stimulate the oxidation steps of the Maillard reaction to increase the production of advanced glycation end products (AGE); when accumulated, these products are considered toxic [294]. The accumulation of AGE is associated with marked upregulation of antioxidant activities [295]. When AGE binds with its receptor, RAGE, an inflammatory state is produced [296-299] via transcription factor NF-kappa B activation followed by cytokine expression [296-298,300,301]. Alcohol may hasten OS through direct and indirect mechanisms that increase apoptosis, alter cell structures, and damage tissue [292]. Additionally, damage to mitochondria coupled with weakened antioxidant defense can incite free radical formation [302,303].

Kalousová et al (2004) reported markedly increased AGE in chronic alcoholics compared to healthy controls [304]. Moreover, their results also supported the notion that AGE production may be prevented or even diminished by antioxidant supplementation with vitamin B derivatives [305,306], and/or vitamins A, C, and E [307]. It is well known that alcoholics often present with a variety of health problems including malnutrition, cachexia, and vitamin deficiencies; all of these states can also promote AGE formation, and further investigation of the possible protective effects of antioxidants is warranted.

In contrast, in vitro studies have demonstrated acetaldehyde to inhibit AGE formation [308]; these results further support those of copious previous studies cardio-protective effects of moderate alcohol intake. Taken together, the effects of alcohol, whether positive or negative, probably depend on the amount consumed [304], since increasing doses can accumulate within tissues and cause irreversible tissue damage, despite future efforts to abstain from alcohol.

Although the effects of alcohol use on female fertility are inconclusive, alcohol has long been known to have negative impacts on the fetus in utero. Mouse embryos exposed to EtOH sustained higher SO anion radical production, lipid peroxidation, and apoptosis, as well as in vitro deformation; however, these toxicities were lessened by simultaneous administration of SOD [309]. Similar results in were found in vivo by Heaton et al [310]. Additionally, Wentzel et al (2006) showed that vitamin E co-administration to EtOH-exposed dams reduced embryo defects and miscarriage [311].

Alcohol consumption has also been related to delayed conception. A Danish study demonstrated an increased risk of infertility in women over the age of 30, who consumed seven or more alcoholic beverages per week. It was concluded that alcohol might exacerbate age-related infertility [312]. The results of this study also support previous reports of dose-related adverse effects of alcohol.

Maternal alcohol use has also been seen to increase the risk for spontaneous abortion and early pregnancy loss [281]. As a pro-oxidant, EtOH use can lead to apoptosis and damage to protective placental systems. Continuous exposure to EtOH in utero [313] could therefore account for the oxidative placental damage implicated in the pathogenesis of pregnancy loss.

Rodent studies have shown a possible association between EtOH and increased placental NOS along with reduced NO within syncytiotrophoblasts, which alters placental blood flow and causes inadequate delivery of nutrients and O_2_ to the fetus. Thus, IUGR is a potential adverse outcome [289,290,313]. The level and length of EtOH exposure are the main factors accounting for alterations in NO production. In low doses, EtOH increases the activities of NO and eNOS, augmenting endothelial vasodilation. On the other hand, higher doses of EtOH can impair endothelial function [289]. Cell damage by NO in vivo results from production of peroxynitrite during NO-SO interaction under oxidative conditions [314]. Hence, NO is considered an important factor contributing to the impaired development of EtOH-exposed fetuses.

### 12.3. Recreational drug use

#### 12.3.1. Cannabinoids

Cannabinoids are active constituents of marijuana, the most commonly used recreational drug used throughout the world. Cannabinoids can generate free radicals which can alter both central and peripheral nervous system functioning [315]. The fundamental component of marijuana known to exert psychological effects in smokers of the drug is known as delta-9-tetrahydrocannabinol (THC) [315]. Endocannabinoid receptors have been detected in female reproductive organs such as the ovary and uterus [273]. Modifications of the endocannabinoid system by exogenous administration of cannabinoid agonists can disturb normal reproductive processes, possibly through free radical production [316].

Delta-9-tetrahydrocannabinol been found to disrupt embryo development and inhibits implantation. Placental transfer of THC accounts for its buildup in reproductive fluids and embryos exposed to THC show affected morphology [317]. Exposure to THC in-utero has been linked with low birth weight [318,319], prematurity, congenital abnormalities, and stillbirth [318,320].

Marijuana use has been shown to disturb hormone patterns and responses, which could explain the elevated risk of primary infertility seen in regular users of the drug compared with non-users [320]. Specifically, the THC component of marijuana may affect female reproduction by hindering oogenesis, inhibiting implantation and embryo development, and may contribute to the culmination of these effects in spontaneous abortion [321].

The generation of ROS [322-324] is often associated with DNA strand breaks induced by THC [315]. Epoxidation of the 9, 10-alkene linkage by THC is the proposed mechanism of DNA damage [325], and DNA alkylatation by epoxides simultaneously generates ROS [324,326].

A study by Sarafian et al (1999) demonstrated significant dose-dependent increases in ROS production in vitro induced by marijuana cigarette smoke containing THC, which was manifested by higher nitrate levels measured in culture, compared with controls. In addition, cigarette smoke lacking THC did not generate increased ROS compared to controls in room air, indicating that heightened ROS production is dependent on the THC component of marijuana smoke [327]. Moreover, according to Sarafian et al, prior studies have shown that chronic marijuana exposure causes a continual decline in GSH antioxidant systems and result in necrotic apoptosis [313], providing further evidence of THC’s cytotoxic effects.

Antioxidants such as vitamin E have been shown to prevent THC-induced neurotoxicity, specifically neuronal cell death [315]. Similarly, antioxidants could potentially inhibit or even reverse the reproductive dysfunction and adverse pregnancy outcomes induced by THC in regular users of marijuana. However, extensive investigation and clinical trials are necessary to determine if antioxidant supplementation is beneficial to reproductive outcomes.

#### 12.3.2. Cocaine

Cocaine has potent stimulant properties that contribute to its highly addictive potential [282] and its use during pregnancy has been linked to adverse outcomes including low birth weight, prematurity [328], IUGR, and miscarriage [273,329,330].

The oxidative pathway of cocaine yields several metabolites that trigger a greater degree of lipid peroxidation than cocaine itself, with simultaneous redox cycling and production of SO and lipid peroxyl radicals [331]. Formaldehyde is one of many oxidative metabolites of cocaine described to generate ROS [7]. Norcocaine is another cocaine metabolite that upon oxidation, is further metabolized to nitroxide [332], which could become toxic if reacted with NO or peroxynitrite [333]. The resultant OS leads to depletion of GSH stores [7].

Undeveloped embryonic and fetal defense systems are unable to counteract an overload of OS without support from exogenous antioxidants [282]. The vasoconstrictive characteristics of cocaine can affect uterine and placental vasculature, subjecting the fetus to hypoxia, as shown in rats by strikingly increased GSSG with acute cocaine exposure and decreased GSH with chronic exposure [334]. Similar alterations in GSH levels were demonstrated by Lee et al (2001), who found a significant dose-dependent reduction in GSH with cocaine exposure and increased inflammatory cytokine production through heightened expression of TNF-alpha and NF-kappa B [335].

Reactive oxygen species can induce apoptosis [336], another outcome associated with the use of cocaine [337,338]. Thiol and deferoxamine were found to prevent against cocaine-induced apoptosis, indicating that ROS influences the apoptosis related to cocaine use [339].

Cocaine has also been shown to induce peroxidative damage to fetal membranes [340], which was found to be offset by vitamin E. Increased lipid peroxidation within the embryos of cocaine-treated mice was observed by Zimmerman et al (1994), and was also found to be prevented by concomitant antioxidant administration [329,341]. In rat embryos, cocaine promoted free radical generation, which halted terminal ET [342].

Taken together, the findings from these studies implicate OS as a contributor to the damage inflicted by cocaine. Cocaine-associated teratogenicity and apoptosis are largely attributed to the OS produced by cocaine metabolites, which are further supported by the demonstrated protective effects of antioxidant. Research investigating potential therapies for cocaine-induced oxidative damage is still underway, and substances such as nitrones, seem promising for trapping the free radicals generated by cocaine metabolites [343] and inhibiting ROS-induced activation of inflammatory pathways [332].

## 13. Environmental and occupational exposures

The stability of reproductive cells and tissues is dependent on balanced concentrations of antioxidants and oxidants [344]. Varied levels of ROS can have both positive and negative impacts on female reproduction. At physiologically appropriate levels, they are involved in cell signaling processes. The excess production of free radicals and subsequent induction of OS, however, have long been known to significantly affect reproductive functions [22]. More recently, environmental pollutants including pesticides have been implicated in the pathogenesis of reproductive disorders [345,346] and infertility. Humans are constantly exposed to pollutants through air, soil, ingestion of contaminated food and water [347]. Mass production of chemicals and their distribution in many consumer goods poses a health threat to the general population through direct and ambient exposure [348].

The 1st trimester of pregnancy carries the highest risk of miscarriage, as it is a critical period of fetal organ development. Affected fetal growth and development during the 2^nd^ trimester may negatively impact 3^rd^ trimester assessment of fetal viability and fetal outcomes. Maternal exposure to various toxins, especially during critical developmental windows can threaten fetal development and produce undesirable outcomes to both the mother and her fetus.

### 13.1. Organochlorine pesticides: DDT

Organochlorines are extensively used in pesticides. They exhibit strong hydrophobic properties and are intensely lipophilic compounds. Organochlorine pesticides (OCPs) are notorious for their toxic effects on nerves [349], but their slow buildup in body tissues of high lipid content over time can negatively impact maternal reproductive abilities as well as the embryo or fetus itself [260]. Elevated levels of many OCPs have been detected in various body compartments such as blood, amniotic fluid, and the placenta [350].

*1, 1, 1-trichloro-2, 2,-bis (4-chlorophenyl)-ethane* (DDT) is an OCP that was widely used as a potent insecticide in the past. In general, incidental human exposure to DDT has been considered relatively non-toxic, but prolonged exposure has long been recognized to adversely affect reproduction [9]. Furthermore, recent reports of even prophylactic exposure have revealed potential for undesirable effects [348]. Although the United States banned the use of DDT in 1973 [351], levels can persist in human body tissue owing to its long half-life of 10-20 years. The accumulation of DDT in body fat and FF may result in exponentially increased levels and toxicity over time [9,347].

In their study, Jirosova et al (2010) were unable to demonstrate affected IVF outcomes in relation to OCP concentrations in FF; however, they did find a two-fold increase in OCP concentrations over time, which may provide a basis for significant reproductive and health concerns. Specifically, it was noted that DDT exposure caused a decrease in diploid oocyte number [9].

Passive maternal exposure to pesticides has been also been demonstrated to increase the risk of miscarriage. An increase in spontaneous abortions was documented in spouses of agricultural male workers who were in direct contact with pesticide chemicals including DDT on a daily basis [352].

### 13.2. Polychlorinated biphenyls

The numerous adverse health effects of polychlorinated biphenyls (PCBs) as constituents of everyday products like makeup, varnish, and pesticides fueled their eradication from the United States market during the latter half of the 1970’s [349]. Like OCPs, PCBs are highly lipophilic, eliciting concern for their persistent presence in the human body secondary to slow degradation even many years after cessation of use.

Human exposure occurs for the most part through consumption of foods containing PCB traces, such as fish, meat, and dairy. Other pathways of exposure may be occupational or via inhalation of surrounding air containing PCB elements [353].

Exposure to PCB has long been implicated as a potential source of reproductive dysfunction (reviewed by [354-359]) and elevated risk of miscarriage. The impact of PCBs on female reproduction has been evidenced by their presence in FF [360-362], ovaries [363], placenta, uterus, and amniotic fluid [364]. PCBs have also been detected within embryos and fetuses [365,366], possibly contributing to their adverse outcomes. Although Meeker et al (2011) [353] and Toft et al (2010) [367] were unable to link PCBs to increased risk for spontaneous abortion, they did document a connection between PCB exposure and failed implantation in IVF cycles, in support of previous studies. These results may substantiate earlier claims relating PCB exposure to decreased fecundability and longer times to conception [353].

Endothelial dysfunction induced by PCBs has also been attributed to increased OS [368-372]. Interestingly, PCB exposure was observed to suppress Vitamin E levels [368,373,374]; since vitamin E and other antioxidants can prevent this endothelial dysfunction, OS is a likely contributor to PCB-associated toxicities [8].

Additionally, PCBs are known to cause cell membrane destruction and increase free radical generation. Although their direct effects on fertility remain unconfirmed, many studies have established their probable role in impairing menses and endometrial quality [9].

### 13.3. Organophosphate pesticides

Oxidative stress has been implicated in undesirable reproductive outcomes induced by organophosphate compounds (OPCs) [375-379]. Studies have found decreased activities and levels of antioxidant enzymes in conjunction with increased lipid peroxide generation [380]. The extent of DNA damage inflicted by OPCs was shown to depend on the amount and length of exposure. Depletion of GSH with concurrently increased ROS generation triggered OS.

The link between OS and DNA damage was further suggested by elevated measurements of the respective biomarkers by Samarawickrema et al (2008), who studied the effects of low-grade long-term exposure to environmental and occupational OPCs [10]. They found significantly increased cord blood MDA levels in samples obtained during spray seasons and increased fetal DNA fragmentation, indicating enhanced fetal OS. Interestingly, maternal OS biomarker levels were unaltered, perhaps due to varied conversion to toxic metabolites or lower maternal metabolic detoxification capacities, both of which can further result in continued OPC accumulation in the placental-fetal compartment, hampered efficacy of antioxidant systems, or altered repair mechanisms.

## 14. Assisted reproductive techniques

Assisted reproductive techniques are advanced technological procedures, which are the treatments of choice in many cases of female and male infertility. They function as an alternative to overcome causative factors of infertility, such as endometriosis, tubal factor infertility, male factor infertility, and are also helpful for women with unexplained infertility [381]. These techniques include intrauterine insemination, IVF, and intracytoplasmic sperm injection (ICSI).

With IVF, sperm-oocyte interaction occurs in culture media, leading to fertilization [142]. Reactive oxygen species may develop as a consequence of increased oocyte number per dish, spermatozoa, and cumulus cell mass. Cumulus cells demonstrate higher antioxidant activity at the beginning of culture than denuded oocytes do [382].

In ICSI, a single sperm is injected into an oocyte’s cytoplasm [142]. It bypasses natural selection, thus allowing for the injection of damaged spermatozoon into the oocyte. Alternatively, the IVF process prevents fertilization by DNA-damaged spermatozoa [383].

**Figure 2:**
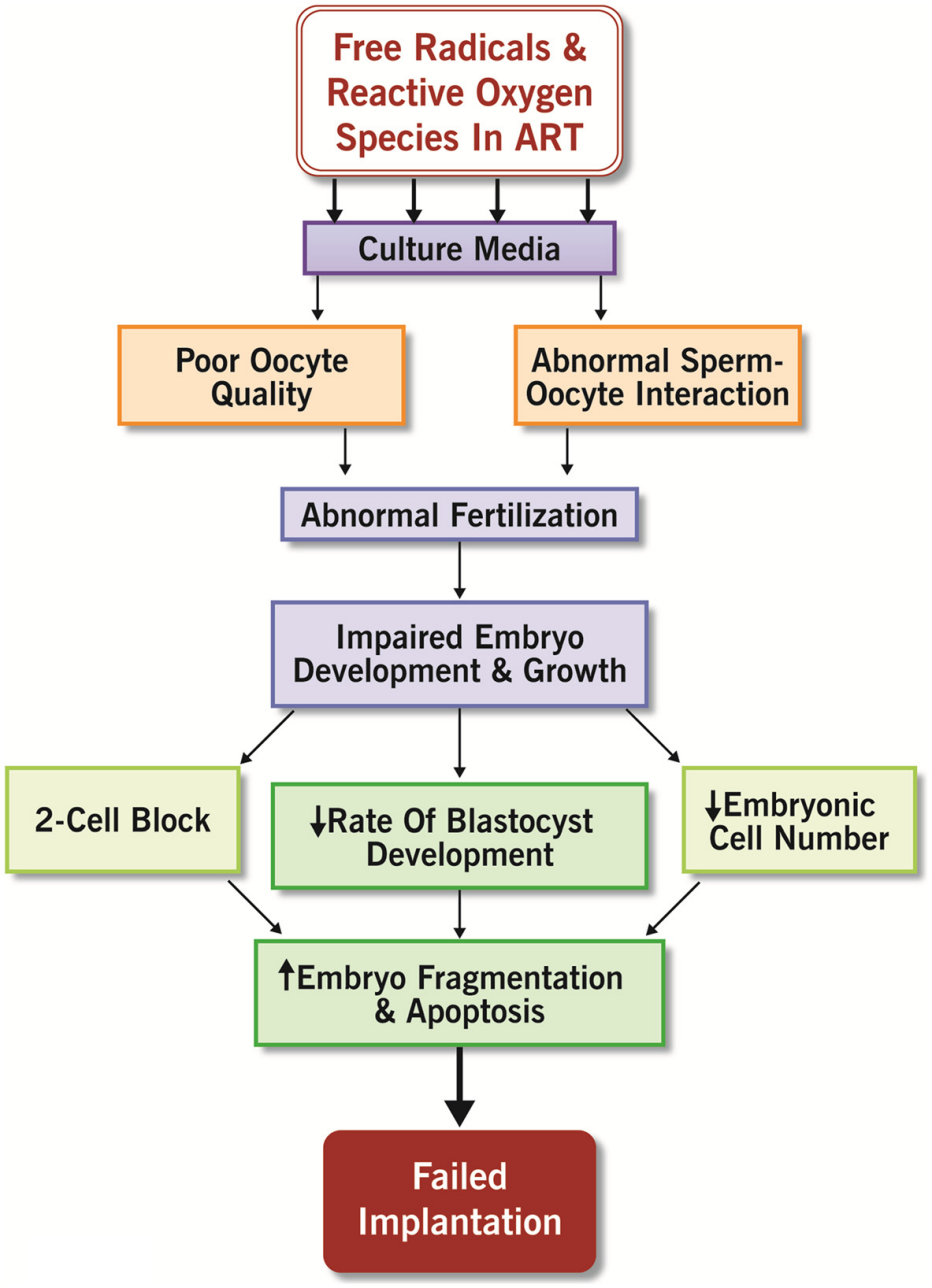
The influence of the presence of free radicals and ROS in ART culture and subsequent effects on embryo development.

Recently, OS has been identified as an important factor in ART success. Oocyte metabolism and a lack of antioxidants combined with the follicular and oviductal fluid of the embryo causes an increase in ROS levels [384]. Follicular fluid is the net result of both the transfer of plasma constituents to follicles and the secretory activity of granulosa and theca cells [385]. The oocyte develops within the FF environment and this intimately affects the quality of oocytes and their interaction with sperm, thus affecting implantation and embryonic development [386] (Figure 2).

Oxidative stress contributes to oocyte quality, and its degree can be assessed by biomarkers of TAC and lipid peroxidation [387]. The effects of OS may be may be further altered by environmental factors. A hyperoxic environment augments SO radical levels by promoting enzyme activity. Particularly in IVF, increased incubation time heightens exposure to O_2_ concentration [43].

As in biological systems, metallic cations act as exogenous sources of OS by stimulating ROS formation in ART culture media, and metal chelators such as EDTA and transferrin can ameliorate the production of ROS [43]. Furthermore, visible light can cause ROS formation, thereby damaging DNA [388]. Fertilization success in ART is determined by the quality of spermatozoa involved [389]. Although ROS contribute to normal sperm functions such as oocyte fusion, capacitation, and acrosome reaction, OS produced by spermatozoa may provoke oxidative damage to the oocyte, decreasing the likelihood for fertilization [18].

The in vitro environment exposes gametes and embryos to an excess of ROS with the absence of enzymatic antioxidant protection normally present during in vivo fertilization and pregnancy. Free radicals are thought to act as determinants in reproductive outcomes due to their effects on oocytes, sperm, and embryos [381]. Oxidative stress disturbs human oocyte intracellular Ca^2+^ homeostasis as well as oocyte maturation and fertilization. During ovulation, ROS are produced within the follicles, however, the excessive production of ROS may increase the risk for poor oocyte quality since oxidative stimulation promotes oocyte maturation and wall rupture within the follicle [390]. Women with > 30% degenerate oocytes demonstrate significantly increased intrafollicular 8-OHdG, indicating DNA damage by OS. However, Tamura et al (2008) found that the administration of melatonin led to a reduction of intrafollicular oxidative damage and a net increase of fertilization and pregnancy rates [391].

A physiologic amount of ROS in FF is indicative of a healthy developing oocyte [392]. Follicular fluid ROS levels of < 107 counted photons per second were significantly negatively correlated with IVF outcome parameters. The culture media can generate ROS at different rates depending on its composition [386]. The impact of OS triggered by culture media can partially deplete oocyte GSH content, enhancing the effect of sustained OS and thus, risking oocyte fertilization and viability [393].

In mice, *sirt3* regulates mitochondrial activity and basal ATP synthesis. It protects against the effects of OS on pre-implantation development under IVF and in vitro culture conditions. A deficiency of *sirt3* can fuel mitochondrial production of ROS, causing activation of p53 and arrested development of pre-implanted embryos [394].

In vitro fertilization can disturb the oxidant-antioxidant balance, rendering the culture media less protected against oxidation. The adverse effects of sustained OS and resulting loss of oocyte antioxidant content were shown to be improved by adding lipophilic and hydrosoluble antioxidants to the culture media to lessen OS [393]. Oral vitamin and mineral supplementation have been shown to increase serum concentrations of GSH and vitamins C and E; these antioxidants have been suggested to play a significant role in IVF outcomes [395].

## 15. Concluding remarks

Oxidative stress is the result of overproduction of ROS in relation to antioxidant defense levels. Excessive ROS production and resulting OS may contribute to aging and several diseased states affecting female reproduction. Endothelial dysfunction secondary to OS contributes to the development of obstetric complications such as early and recurrent pregnancy loss, preeclampsia, IUGR, and preterm labor. Reactive oxygen and nitrogen species can negatively affect embryo implantation and may influence the development of reproductive disorders such as endometriosis and preeclampsia. Although the pathogenesis of preeclampsia has yet to be determined, placental ischemia/hypoxia is regarded as an important contributor through the induction of OS, which in turn can trigger the endothelial cell dysfunction characteristic of the disease. Altered vasomotor functions have been demonstrated by failed embryo implantation and reduced placental perfusion in preeclampsia and endometriosis. These effects have been reported to improve with the aid of antioxidants, and thus could minimize the associated risk for infertility.

Extremes of body weight have been shown to negatively affect the fecundability of females and adversely affect fetuses and embryos through oxidative mechanisms. Moderate exercise may assist obese women reduce weight and restore their fertility. Lifestyle factors such as maternal smoking, alcohol consumption, and recreational drug use stimulate production of unfavorable amounts of ROS leading to OS, which renders physiological processes of female reproduction and the fetus vulnerable to oxidant-induced damage. Exposure to environmental pollution can also give rise to excessive OS during pregnancy, and has increasingly raised concern about the impact of pollutant exposure on maternal and fetal health.

The effects of free radicals on oocytes, sperm, and embryos have been implicated in poor reproductive outcomes in ART. The in vitro environment subjects gametes and embryos to an abundance of ROS in the absence of enzymatic antioxidant defenses that are normally present during in vivo fertilization and pregnancy. Ideally, ART success may be attained if in vivo conditions are sufficiently imitated. To this effect, several studies have shown that antioxidant supplementation of the culture media may improve pregnancy outcomes.

In spite of the perceived hypotheses regarding the benefits of antioxidant supplementation on pregnancy outcomes, clinical trials investigating the use of antioxidants to treat reproductive disorders have reported largely conflicting results. Moreover, the bulk of evidence in support of therapeutic effects of antioxidants to date, have been observed through experimental studies on animals or through in vitro studies. In the future, human clinical trials will help to clarify the efficacy of antioxidants as potential therapies for infertility.

## 16. Abbreviations

8-iso-PGF2-alpha: 8-iso-prostaglandin F2-alpha; 8-OHdG: 8-oxodeoxyguanosine; 8-OxOdG: 8-oxo-7,8- dihydro-2-deoxyguanosine; AGE: Advanced glycation end-products; ART: Assisted reproductive techniques; ASK: Apoptosis signaling regulation kinase; AT1-AA: Autoantibodies against AT1 receptor; ATP: Adenine triphosphate; BMI: Body mass index; [Ca^2+^]_i_: Intracellular calcium concentration; CCE: Capacitative calcium entry; cGMP: Cyclic guanosine monophosphate; CRP: (C-reactive protein); CSH: Cysteamine; Cu: Copper; DDT: 1, 1, 1-trichloro-2, 2,-bis (4-chlorophenyl)-ethane; EDTA: Ethylenediamine tetra-acetic acid; eNOS/NOS III: Endothelial nitric oxide synthase; ERK 1/2: Extracellular regulated kinase; ET: Electron transfer; ETC: Electron transport chain; EtOH: Ethanol; ER: Endoplasmic reticulum; Fe^2+/3+^: Iron; FF: Follicular fluid; FSH: Follicular stimulating hormone; GPx: Glutathione peroxidase; GSH: Glutathione; GSSG: Oxidized glutathione; hCG: Human chorionic gonadotropin; HDL: High-density lipoprotein; HIF: Hypoxia-inducible factor; HO: Heme oxygenase; H_2_O: Water; H_2_O_2_: Hydrogen peroxide; HRT: Hormone replacement therapy; HSP: Heat shock protein; ICSI: Intracytoplasmic sperm injection; IL: Interleukin; iNOS/NOS II: Inducible nitric oxide synthase; IUGR: Intrauterine growth restriction; IVF: In-vitro fertilization; JNK: c-Jun *N*-terminal kinases; LH: Luteinizing hormone; MAPK: Mitogen-activated protein kinases; MDA: Malondialdehyde; Mn: Manganese; MTHFR: Methyl-tetra-hydrofolate reductase; NAC: N-acetylcysteine; NADPH: Nicotinamide adenine dinuleotide phosphate; NK: Natural killer; NOS: Nitric oxide synthase; nNOS/nNOS I: Neuronal nitric oxide synthase; NO: Nitric oxide; NO_2_: Nitrogen dioxide; O_2_: Oxygen; OCPs: Organochlorine pesticides; OH^*^: Hydroxyl radical; ONOO^−^: Peroxynitrite; OPCs: Organophosphate compounds; OS: Oxidative stress; oxLDL: Oxidized low-density lipoprotein; PCBs: Polychlorinated biphenyls; PCOS: Polycycstic ovary syndrome; PG: Prostaglandin; PON 1: Paraoxonase-1; RNS: Reactive nitrogen species; ROS: Reactive oxygen species; RPL: Recurrent pregnancy loss; Se: Selenium; sFlt-1: Soluble receptor for vascular endothelial growth factor; SHBG: Sex hormone binding globulin; SO: Superoxide; SOD: Superoxide dismutase; STBM: Syncytiotrophoblast microvillus membrane; TAC: Total antioxidant capacity; TAS: Total antioxidant status; TBARS: Thiobarbituric acid reactive substances; THC: Delta-9-tetrahydrocannabinol; TLR: Toll-like receptor; TNF: Tumor necrosis factor; Trx: Thioredoxin; VEGF: Vascular endothelial growth factor; Zn: Zinc.

## 17. Competing interests

The authors declare that they have no competing interests.

## 18. Authors’ contributions

All of the authors contributed to the conception of the review. AAM, BJP, and AS performed literature searches and selected the studies and reviews discussed in the manuscript. The first draft of the manuscript was also prepared by AAM, BJP, and AS. AAM and BJP performed subsequent amendments. BJP performed further in depth interpretations of the discussed studies and provided critical insights throughout the manuscript. BJP reviewed and finalized the manuscript. All authors read and approved the final manuscript.
